# Sex differences in cardiac risk and kidney function: serum creatinine versus cystatin C

**DOI:** 10.1186/s12916-025-04588-9

**Published:** 2025-12-30

**Authors:** Nicole L. De La Mata, James A. Hedley, Angela C. Webster, Michael K. Sullivan, Brenda M. Rosales, Patrick B. Mark, Jennifer S. Lees

**Affiliations:** 1 Sydney School of Public Health, Faculty of Medicine and Health, The University of Sydney, Camperdown, NSW Australia; 2 Centre for Renal and Transplant Research, Westmead Hospital, Sydney, NSW Australia; 3 School of Cardiovascular and Metabolic Health, University of Glasgow, Glasgow, UK

**Keywords:** Sex, Cardiovascular risk, Kidney function, Creatinine, Cystatin C

## Abstract

**Background:**

Cystatin C may better assess cardiovascular risk than serum creatinine for kidney function, but its accuracy may vary by sex. We evaluated sex differences in cardiac risk using estimated kidney function from either biomarker.

**Methods:**

We included all adults from the UK Biobank without prior cardiac event who had kidney function and baseline data. We defined cardiac events and deaths using ICD-10 codes in hospital or death records. We fitted cause-specific Cox models to evaluate sex differences in cardiac outcomes using estimated glomerular filtration (mL/min/1.73m^2^) from serum creatinine (eGFRCr), cystatin C (eGFRCys) and both (eGFRCr-Cys).

**Results:**

Among 394,920 adults (55% female), 19,689 (9%) females and 28,540 (16%) males had cardiac events. In adjusted models, eGFRCys and eGFRCr-Cys showed stronger associations with increased cardiovascular risk in females than when using eGFRCr (*p* < 0.001). Females with eGFRCys 45–59 had elevated cardiac risk (HR 1.08, 95% CI 1.03–1.14) compared to males with eGFRCys 90–104—an effect not seen with eGFRCr or eGFRCr-Cys. eGFRCr showed a J-shaped association with cardiac risk, being increased in males but reduced in females when eGFRCr ≥ 105 (females: HR 0.65, 95% CI 0.61–0.69; males: HR 1.18, 95% CI 1.12–1.24 versus males with eGFRCr 90–104). The risk of cardiac events was more linear in adjusted models with eGFRCys.

**Conclusions:**

Measurement of cystatin C improves estimation of cardiac risk associated with kidney function, particularly for females. Incorporating eGFRCys, rather than eGFRCr, into cardiovascular risk assessment may be more important for early detection and management of high-risk females with CKD.

**Supplementary Information:**

The online version contains supplementary material available at 10.1186/s12916-025-04588-9.

## Lay summary 

Kidney function estimates from cystatin C provide a better assessment of cardiac risk by sex than when estimated from serum creatinine, especially for women at high risk.

- When using cystatin C for kidney function, the relationship with cardiac risk was more consistent for both sexes, being lower risk with better kidney function.
- Women with mildly reduced kidney function based on cystatin C had a higher risk of cardiac events, an effect not seen when using creatinine.

## Background

Cardiovascular disease is the leading cause of death globally, projected to account for over 23.6 million deaths per year by 2030 [1]. People with chronic kidney disease (CKD) are an under-recognised high-risk group for cardiovascular disease. Kidney disease is an independent risk factor for cardiovascular disease, and exposure to chronic dialysis further increases the risk of cardiac events [2]. In the UK, more than one in five adults with cardiovascular disease are estimated to have CKD [3]. Despite this, kidney disease is often overlooked in cardiovascular risk appraisal. National and international guidelines for cardiovascular risk management often do not recognise CKD as a notable risk factor, and many cardiovascular risk-assessment tools overlook kidney function [4], although some have been recently revised [5–7].

Cardiovascular risk increases with advancing CKD and so estimated glomerular filtration rate (eGFR) is a predictor of cardiovascular risk. eGFR is calculated using both patient factors and serum biomarkers [8], namely serum creatinine, cystatin C or both. Cystatin C provides stronger cardiovascular risk assessment than creatinine, likely due in part to its non-GFR determinants that seemingly capture aspects of cardiometabolic risk [9].

Both creatinine and cystatin C, and their association with the risk of adverse events, have the potential to be associated with substantial sex differences. Serum creatinine is released primarily from muscle, and serum levels are mostly driven by muscle mass. In individuals with lower-than-average muscle mass, eGFR based on serum creatinine may overestimate true GFR and, therefore, underestimate the association between lower GFR and the risk of adverse outcomes. Females generally have lower muscle mass and lose muscle at a slower rate with age than males [10]. On the other hand, pre-menopausal females typically have lower rates of cardiometabolic risk factors and disease than males. These risk factors are less important determinants of cardiovascular disease outcomes in females than males [11]. It is therefore plausible that the association of cardiovascular risk with eGFR may differ according to the biomarker used. We sought to evaluate (i) the modifying role of sex between the relationship of kidney measures and cardiovascular events, and (ii) the added value of eGFRCr, eGFRCys and eGFRCr-Cys to baseline cardiovascular risk models to determine if there are differences in performance between females and males.

## Methods

### Study design and setting

We used data from the UK Biobank, a large prospective cohort study of adults aged 40–69 years recruited during 2006–2010. A more detailed study description and protocol has been published elsewhere [12, 13]. Briefly, an invitation to participate was sent to all adults aged 40–69 years registered with the UK National Health Service and living within 25 miles from any of the 22 study assessment centres. A total of 503,325 adults consented and were recruited. At baseline assessment, participant data collection included a questionnaire on lifestyle, environment and medical history; physical examination measures; and biological samples. Follow-up for all participants included data linkage to routine administrative health data from the UK National Health Service, such as mortality, cancer registry, hospitalisations and primary care data. For a subset of approximately 25,000 participants, baseline assessment was repeated every few years.

### Participants

Our study population included all people who had not previously had a cardiac event, had available biochemistry measures of serum creatinine and serum cystatin C and had relevant clinical data at baseline to estimate kidney function. A cardiac event included arrhythmia, ischaemic heart disease, heart failure, valvular disease, and cardiomyopathy.

This study received ethics approval from the North-West Multi-Centre Research Ethics Committee (REC reference 11/MW/03820) and was conducted under UK Biobank project code 69,891.

### Clinical variables

Clinical variables were defined using a combination of self-report and International Classification of Disease and Related Health Problems (ICD) 10th Revision 2016 (ICD-10) codes from administrative health records. Self-reported measures only included medical history of cardiovascular disease and diabetes, smoking history and ethnicity.

The baseline eGFR was calculated using a single baseline measure [14] of serum creatinine (eGFRCr) [15], cystatin C (eGFRCys) [16] or a combination of both serum creatinine and cystatin C (eGFRCr-Cys) [16], based on CKD-EPI Eqs. 2009 which is currently recommended for UK clinical practice [17]. eGFR was categorised into the following: < 30 (indicating worst kidney function), 30–44, 45–59, 60–74, 75–89, 90–104 (indicating normal kidney function), ≥ 105 mL/min/1.73m^2^. For comparative analyses between sex and eGFR categories, males with eGFR 90–104 mL/min/1.73m^2^ were considered the reference group. We selected males with 90–104 eGFR as the reference group across all our analyses as this is the expected healthy range for people in our study population, and the most common group [18]. We further reported on the association with the ratio of eGFRCys/eGFRCr < 1.

All blood and spot urine samples, including creatinine and cystatin C, were analysed at a central laboratory [19]. Serum and urine creatinine were measured using an enzymatic, IDMS-traceable method on the Beckman Coulter AU5800 instrument. Serum cystatin C was measured using a latex-enhanced immunoturbidimetric method on the Siemens ADVIA 1800 instrument. Each assay was registered with an external quality assurance scheme, and assay performance was externally verified via the results returned from participation in these schemes.

### Outcomes

There were two main outcomes of interest: (1) any cardiac events (composite of cardiac event and cardiac deaths); and (2) any cardiac deaths. All-cause cardiac events and deaths were defined by ICD-10 codes listed in hospital episodes and causes of death, including arrhythmia (I44–I49), ischaemic heart disease (I20–I25), heart failure (I50), valvular disease (I34–I37) and cardiomyopathy (I42). The UK Biobank has a validated algorithm for myocardial infarction [20]. While there is no validated algorithm for cardiac events and deaths in UK Biobank, we used ICD-10 codes commonly used to report on major cardiovascular events from administrative health databases [21]. We measured time at risk from the date of enrolment into UK Biobank until the earliest of the outcome of interest, other deaths, or censor date (31st August 2020).

### Statistical analyses

Patient characteristics were summarised overall and by sex, using absolute counts and proportions for categorical variables and median and interquartile interval (IQI) for continuous variables.

We estimated the cumulative incidence of cardiac events and death, stratified by sex, and adjusting separately for each of eGFRCr, eGFRCys and eGFRCr–Cys. The Aalen–Johansen estimator was used for cardiac events only and for cardiac death, considering competing risks for non-cardiac death.

We applied cause-specific Cox proportional hazards models to evaluate whether the association with the cardiac outcomes of interest varied by sex and eGFR using interaction terms. Separate models were fitted for each eGFRCr, eGFRCys and eGFRCr–Cys, and interaction terms with sex were fitted in both unadjusted and adjusted models. We further examined the following: (1) a three-way interaction with sex, eGFR and age < 50 years versus age ≥ 50 years; and (2) a two-way interaction with sex and eGFRCys/eGFRCr < 1 compared to eGFRCys/eGFRCr ≥ 1.

Unadjusted models included main and interaction effects between sex and eGFR only. Adjusted models further included characteristics: age at recruitment (years), smoking history (current, former and never), baseline comorbidities (any of diabetes, cancer history and hypertension—either self-reported or based on medication use), ratio of total to HDL cholesterol, systolic blood pressure and medication use for cholesterol. We performed these models for all-cause and subtype cardiac outcomes of interest.

Model performance when using eGFRCr, eGFRCys and eGFRCr–Cys for the same cardiac outcome of interest was assessed using the following: the C-statistic, being the area under the curve where higher values indicated better discrimination; AIC, representing the model fit where lower values indicated better model fit; and calibration plots, comparing the predicted survival probability against the observed survival outcome at 10 years follow-up.

Data were analysed using Stata 16 [22] and R Statistical Software version 4.4.2 using RStudio [23].

## Results

### Study characteristics

**Fig. 1:**
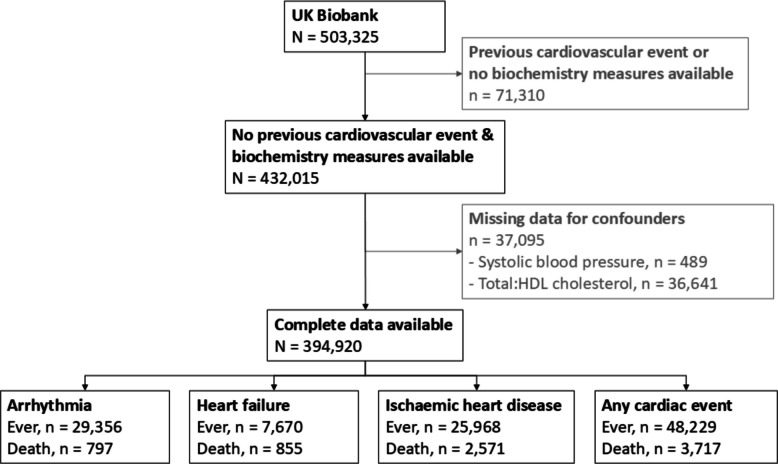
Flowchart of UK Biobank participants included in the analysis

A total of 394,920 people were included in our study, comprising 218,988 (55%) females and 175,932 (45%) males (Fig. 1). Overall, females and males had a median age of 57 years (IQI 50, 63 in females; 49, 63 in males), but males had a higher proportion of current/former smokers, those with diabetes or hypertension, and a higher median total:HDL cholesterol ratio (females 3.7, IQI 3.1, 4.4; males 4.4, IQI 3.7, 5.3; Table 1).

**Table 1:** Baseline characteristics of UK Biobank participants included in our study

	**Female**		**Male**		**Total**	
**Characteristics, n (column %)**	*N* = 218,988		*N* = 175,932		*N* = 394,920	
**Follow-up years, median (IQI)**	11.6	(10.9–12.3)	11.6	(10.9–12.3)	11.6	(10.9–12.3)
**Age, median (IQI)**	57	(50–63)	57	(49–63)	57	(50–63)
<40	1	(<1)	6	(<1)	7	(<1)
40–49	53,347	(24)	44,596	(25)	97,943	(25)
50–59	76,297	(35)	58,170	(33)	134,467	(34)
60+	89,343	(41)	73,160	(42)	162,503	(41)
**Smoker**						
Never	131,943	(60)	89,411	(51)	221,354	(56)
Former	67,861	(31)	64,654	(37)	132,515	(34)
Current	19,184	(9)	21,867	(12)	41,051	(10)
**Comorbidities**						
Diabetes	7,208	(3)	9,935	(6)	17,143	(4)
Cancer	19,047	(9)	10,154	(6)	29,201	(7)
Hypertension	50,526	(23)	49,385	(28)	99,911	(25)
Hyperlipidaemia	22,534	(10)	28,921	(16)	51,455	(13)
**Systolic blood pressure (mmHG), median (IQI)**	135	(122–150)	141	(130–154)	138	(126–152)
**Total:HDL cholesterol, median (IQI)**	3.7	(3.1–4.4)	4.4	(3.7 - 5.3)	4.0	(3.3 - 4.8)
**eGFRCr**						
<30	114	(<1)	152	(<1)	266	(<1)
30–44	468	(<1)	407	(<1)	875	(<1)
45–59	3,725	(2)	2,513	(1)	6,238	(2)
60–74	10,889	(5)	7,772	(4)	18,661	(5)
75–89	70,356	(32)	59,247	(34)	129,603	(33)
90–104	103,831	(47)	82,945	(47)	186,776	(47)
105+	29,605	(14)	22,896	(13)	52,501	(13)
**eGFRCys**						
<30	193	(<1)	225	(<1)	418	(<1)
30–44	1,060	(<1)	884	(<1)	1,944	(<1)
45–59	7,464	(3)	5,290	(3)	12,754	(3)
60–74	18,302	(8)	14,399	(8)	32,701	(8)
75–89	78,631	(36)	70,393	(40)	149,024	(38)
90–104	77,793	(36)	53,671	(31)	131,464	(33)
105+	35,545	(16)	31,070	(18)	66,615	(17)
**eGFRCr-Cys**						
<30	142	(<1)	179	(<1)	321	(<1)
30–44	526	(<1)	524	(<1)	1,050	(<1)
45–59	3,681	(2)	2,504	(1)	6,185	(2)
60–74	11,319	(5)	8,234	(5)	19,553	(5)
75–89	80,555	(37)	69,224	(39)	149,779	(38)
90–104	85,979	(39)	70,093	(40)	156,072	(40)
105+	36,786	(17)	25,174	(14)	61,960	(16)

Females and males had a similar distribution across eGFRCr categories, where nearly half (47%) had > 90–105 mL/min/1.73m^2^, approximately a third had > 75–90 mL/min/1.73m^2^ and less than 2% had < 60 mL/min/1.73m^2^. However, when based on eGFRCys, 69,661 (32%) females and 58,742 (33%) males were reclassified into lower ranges, and 45,069 (21%) females and 37,677 (21%) males were reclassified into higher ranges. Using eGFRCys, those with < 60 mL/min/1.73m^2^ remained the minority (< 4%), but represented an extra 4410 females and 3327 males.

There were 19,689 (9%) females and 28,540 (16%) males who had all-cause cardiac events and 1065 (< 1%) females and 2652 (2%) males who had all-cause cardiac deaths (Table 2) over a total follow-up of 2,533,911 person-years in females (median follow-up of 11.6 years, IQI 10.9, 12.3 years) and 2,028,815 person-years in males (median follow-up of 11.6 years, IQI 10.9, 12.3 years).

**Table 2:** Total number of cardiovascular events and deaths in females and males

	**Female** *N* = 218, 988		**Males** *N* = 175,932		**Total** *N* = 394,920	
	*N*	(%)	*N*	(%)	*N*	(%)
**Any cardiovascular event**	19,689	(9)	28,540	(16)	48,229	(12)
Arrhythmia	12,118	(6)	17,238	(10)	29,356	(7)
Heart failure	3104	(1)	4,566	(3)	7670	(2)
Ischaemic heart disease	9608	(4)	16,360	(9)	25,968	(7)
Cardiomyopathy	650	(<1)	852	(<1)	1502	(<1)
Valvular disease	704	(<1)	959	(<1)	1663	(<1)
**Cardiovascular death**^a^	1065	(<1)	2652	(2)	3717	(<1)
Arrhythmia	274	(<1)	523	(<1)	797	(<1)
Heart failure	291	(<1)	564	(<1)	855	(<1)
Ischaemic heart disease	620	(<1)	1951	(1)	2571	(<1)
Cardiomyopathy	33	(<1)	88	(<1)	121	(<1)
Valvular disease	70	(<1)	153	(<1)	223	(<1)

^a^Deaths may be assigned to multiple cardiac events

**Fig. 2:**
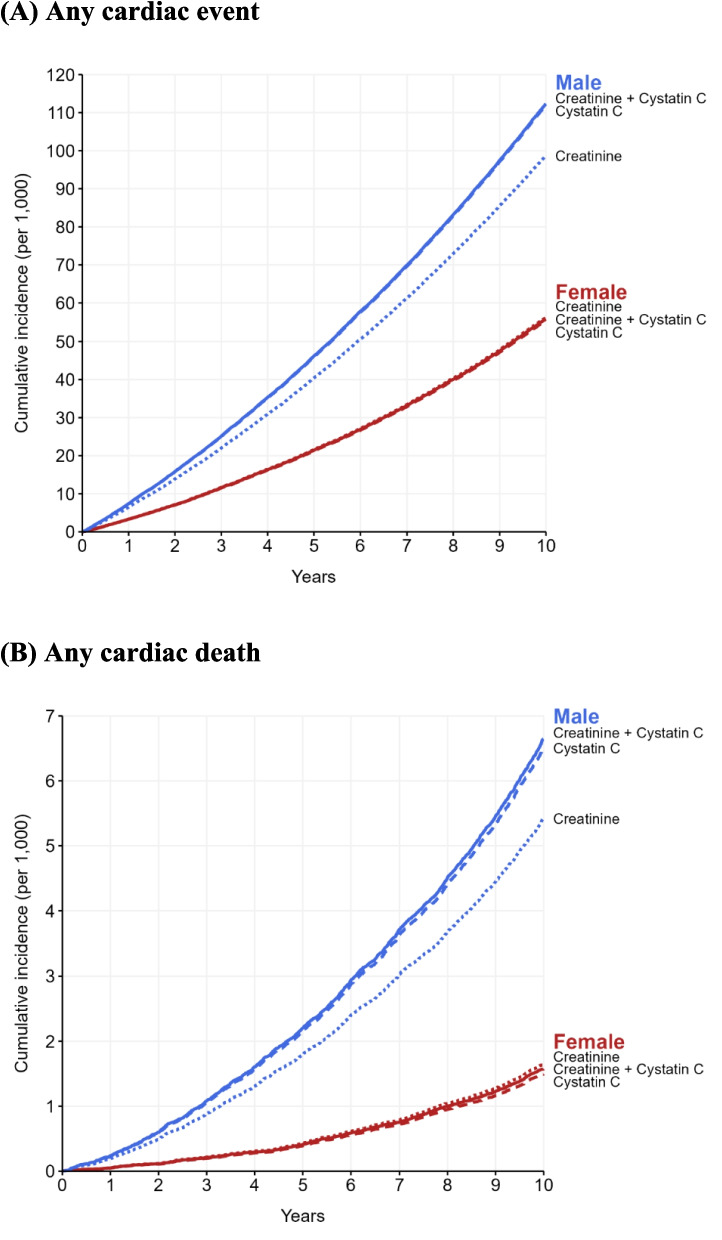
Cumulative incidence per 1000 person-years of **A** any cardiac event and **B** any cardiac death by sex and eGFR (Creatinine, Cystatin C and Creatinine and Cystatin C)

Cumulative incidence of any cardiac events and death by sex and eGFRCr, eGFRCys and eGFRCr-Cys is given in Fig. 2. This was consistently higher in males, and more so using eGFRCys or eGFRCr-Cys.

### All-cause cardiac events

Sex and eGFR were significant modifiers in adjusted models for any cardiac event (interaction term for sex with the following: eGFRCr = *p* < 0.01; eGFRCys = *p* < 0.001; eGFRCr-Cys = *p* < 0.001). Model estimates for all covariates for unadjusted models are given in Additional File 1: Table S1, and adjusted models are given in Additional File 1: Table S2.

**Fig. 3:**
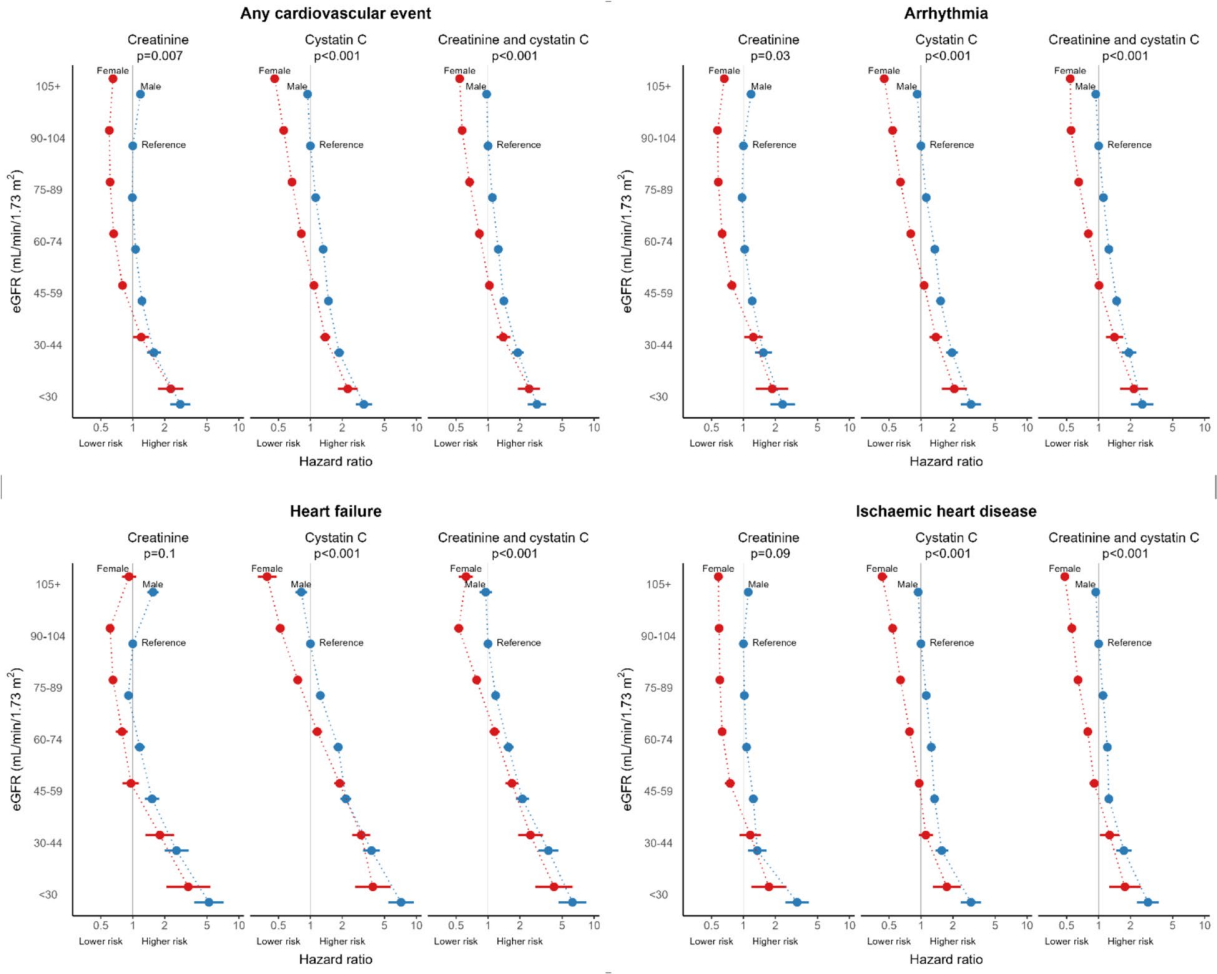
Forest plots of time to any cardiac death and cardiac subtype death by eGFR and sex. eGFR is measured in mL/min/1.73 m^2^. *P*-values are for the interaction between eGFR and sex

The risk of cardiac event increased consistently with lower eGFRCr, eGFRCys and eGFRCr-Cys for both sexes (Fig. 3, Table 3). However, males had a greater risk of cardiac event compared to females at lower eGFR. The risk was highest in the adjusted model with eGFRCys, where for eGFRCys < 30 mL/min/1.73m^2^ males had three times the risk (HR 3.19, 95% CI 2.67, 3.82) and females had two times the risk (HR 2.25, 95% CI 1.81, 2.79) of cardiac event compared to males with eGFRCys 90–104 mL/min/1.73m^2^.

**Table 3:** Adjusted models for analysis of time to first cardiac event and cardiac death

		**Events**				**Deaths**			
**eGFR and sex**		*N*	HR	95% CI	*p*	*N*	HR	95% CI	*p*
**eGFR (creatinine)**					0.007				0.06
Female	*<30*	51	2.28	(1.73, 3.00)		15	5.99	(3.59, 9.99)	
	*30–44*	137	1.20	(1.01, 1.42)		23	1.93	(1.27, 2.92)	
	*45–59*	682	0.80	(0.74, 0.87)		57	0.74	(0.57, 0.97)	
	*60–74*	1448	0.66	(0.63, 0.70)		98	0.55	(0.45, 0.68)	
	*75–89*	7170	0.61	(0.59, 0.63)		355	0.40	(0.35, 0.45)	
	*90–104*	8957	0.60	(0.59, 0.62)		448	0.41	(0.36, 0.46)	
	*105+*	1244	0.65	(0.61, 0.69)		69	0.65	(0.51, 0.84)	
Male	*<30*	83	2.80	(2.25, 3.47)		22	5.03	(3.29, 7.68)	
	*30–44*	171	1.58	(1.36, 1.84)		45	3.43	(2.54, 4.64)	
	*45–59*	774	1.22	(1.14, 1.32)		114	1.70	(1.40, 2.06)	
	*60–74*	1816	1.06	(1.00, 1.11)		197	1.20	(1.03, 1.41)	
	*75–89*	10,975	0.99	(0.96, 1.01)		971	0.98	(0.90, 1.07)	
	*90–104*	12,620	ref	-		1079	ref	-	
	*105+*	2101	1.18	(1.12, 1.24)		224	1.80	(1.55, 2.11)	
**eGFR (cystatin C)**					<0.001				<0.001
Female	*<30*	83	2.25	(1.81, 2.79)		30	7.66	(3.53, 16.61)	
	*30–44*	337	1.38	(1.23, 1.54)		55	1.15	(0.50, 2.63)	
	*45–59*	1735	1.08	(1.03, 1.14)		168	1.44	(0.99, 2.07)	
	*60–74*	2891	0.82	(0.78, 0.85)		194	0.98	(0.70, 1.36)	
	*75–89*	8381	0.67	(0.65, 0.70)		410	0.57	(0.43, 0.75)	
	*90–104*	5197	0.56	(0.54, 0.58)		173	0.35	(0.24, 0.50)	
	*105+*	1065	0.46	(0.43, 0.49)		35	0.13	(0.04, 0.41)	
Male	*<30*	124	3.19	(2.67, 3.82)		41	8.61	(4.32, 17.19)	
	*30–44*	386	1.87	(1.68, 2.07)		95	3.97	(2.43, 6.51)	
	*45–59*	1752	1.48	(1.40, 1.56)		273	2.55	(1.83, 3.55)	
	*60–74*	3822	1.32	(1.26, 1.37)		455	1.95	(1.46, 2.59)	
	*75–89*	12,888	1.12	(1.09, 1.15)		1102	1.10	(0.86, 1.41)	
	*90–104*	6833	ref	-		523	ref	-	
	*105+*	2735	0.94	(0.90, 0.98)		163	0.84	(0.55, 1.28)	
**eGFR (creatinine and cystatin C)**					<0.001				<0.001
Female	*<30*	67	2.44	(1.91, 3.10)		21	6.74	(2.74, 16.57)	
	*30–44*	173	1.39	(1.20, 1.62)		30	1.37	(0.51, 3.73)	
	*45–59*	889	1.03	(0.96, 1.11)		98	1.20	(0.75, 1.91)	
	*60–74*	1938	0.83	(0.79, 0.87)		131	0.90	(0.63, 1.30)	
	*75–89*	9107	0.67	(0.65, 0.69)		495	0.64	(0.50, 0.81)	
	*90–104*	6057	0.57	(0.56, 0.59)		223	0.36	(0.26, 0.49)	
	*105+*	1458	0.54	(0.51, 0.57)		67	0.34	(0.18, 0.65)	
Male	*<30*	99	2.89	(2.37, 3.53)		28	4.90	(2.00, 12.04)	
	*30–44*	241	1.91	(1.68, 2.17)		62	4.02	(2.26, 7.17)	
	*45–59*	868	1.41	(1.31, 1.51)		145	2.16	(1.44, 3.24)	
	*60–74*	2287	1.25	(1.19, 1.31)		310	2.14	(1.60, 2.85)	
	*75–89*	13,621	1.10	(1.07, 1.13)		1225	1.11	(0.90, 1.38)	
	*90–104*	9297	ref	-		720	ref	-	
	*105+*	2127	0.97	(0.92, 1.01)		162	0.91	(0.58, 1.43)	

eGFR is measured in mL/min/1.73m^2^. *P*-values are for the interaction between eGFR and sex

When using eGFRCr or eGFRCr-Cys, males with eGFR 45–59 mL/min/1.73m^2^ were at higher risk of cardiac events, but females were not, compared to the reference of males with eGFR 90–104 mL/min/1.73m^2^. By comparison, females with eGFRCys 45–59 mL/min/1.73m^2^ had an increased risk of cardiac events by 8% (HR 1.08, 95% CI 1.03, 1.14) compared to males with eGFRCys 90–104 mL/min/1.73m^2^.

There was a J-shaped association between eGFRCr and cardiac risk in both males and females. Compared to males with eGFRCr 90–104 mL/min/1.73m^2^, the risk of cardiac event was increased in males, but reduced in females, when eGFRCr ≥ 105 mL/min/1.73m^2^ (females: HR 0.65, 95% CI 0.61, 0.69; males: HR 1.18, 95% CI 1.12, 1.24). The risk of cardiac event was more linear in adjusted models with eGFRCys (Fig. 3); the lowest risk of cardiac event was seen at the highest category of eGFRCys in both males and females.

Model performance based on AIC and C-statistic was similar between all eGFR models, although eGFRCys had the best model performance based on the lower AIC (Additional File 1: Table S3). Similarly, the calibration plot did not demonstrate major deviations from the line of equality; however, eGFRCr models did have more frequent minor deviations (Additional File 2: Figure S1).

The three-way interaction term with age < 50 years compared to age ≥ 50 years was significant for eGFRCr models only; however, the fewer events per stratum resulted in large confidence intervals and little to no noteworthy differences in eGFR effect by sex and age. The two-way interaction term with eGFRCys/eGFRCr ratio and sex was significant (*p* < 0.001). Females with eGFRCys/eGFRCr < 1 (HR 1.33, 95% CI 1.29, 1.37) had a higher risk of cardiac events than their male counterparts with eGFRCys/eGFRCr < 1 (HR 1.19, 95% CI 1.16, 1.22).

The relationship between sex and eGFR did vary by cardiac subtype event (Table 4, Fig. 3, Additional File 1: Table S1, Additional File 1: Table S2). Sex and eGFRCr were not effect modifiers for heart failure and ischaemic heart disease, indicating that the risk of these cardiac events for different eGFRCr values did not vary by sex. However, sex and eGFRCys or eGFRCr-Cys were effect modifiers for all subtypes. Similar to the overall results, the risk of arrhythmia, heart failure or ischaemic heart disease was increased in males with eGFRCr ≥ 105 mL/min/1.73 m^2^ but was not when eGFRCys or eGFRCr-Cys was ≥ 105 mL/min/1.73m^2^. Compared to males with eGFR 90–104 mL/min/1.73m^2^, males with eGFRCr, eGFRCys and eGFRCr-Cys 45–59 and 60–74 mL/min/1.73m^2^ were at higher risk of arrhythmia and heart failure events. In females, excess risk at the same levels of eGFR was only detected using eGFRCys.

**Table 4:** Adjusted models for analysis of time to first cardiac subtype event and cardiac subtype death

		**Events**			**Deaths**		
**eGFR and sex**		HR	95% CI	*p*	HR	95% CI	*p*
**(A) Arrhythmia**							
**eGFRCr**				0.03			0.6
Female	< *30*	1.87	(1.31, 2.66)		6.66	(2.47, 18.01)	
	*30–44*	1.24	(1.02, 1.52)		0.73	(0.18, 2.96)	
	*45–59*	0.78	(0.70, 0.85)		0.64	(0.35, 1.18)	
	*60–74*	0.63	(0.59, 0.68)		0.69	(0.46, 1.03)	
	*75–89*	0.58	(0.56, 0.60)		0.55	(0.43, 0.70)	
	*90–104*	0.57	(0.55, 0.59)		0.50	(0.40, 0.63)	
	*105* +	0.66	(0.61, 0.72)		0.46	(0.22, 0.94)	
Male	< *30*	2.34	(1.80, 3.06)		4.96	(2.03, 12.10)	
	*30–44*	1.54	(1.28, 1.86)		3.18	(1.62, 6.24)	
	*45–59*	1.21	(1.10, 1.32)		1.56	(1.00, 2.44)	
	*60–74*	1.03	(0.97, 1.10)		1.33	(0.96, 1.84)	
	*75–89*	0.97	(0.94, 1.01)		1.05	(0.86, 1.28)	
	*90–104*	ref	-		ref	-	
	*105* +	1.18	(1.11, 1.27)		1.4	(0.91, 2.15)	
**eGFRCys**				< 0.001			0.07
Female	< *30*	2.06	(1.57, 2.70)		7.66	(3.53, 16.61)	
	*30–44*	1.38	(1.20, 1.58)		1.15	(0.50, 2.63)	
	*45–59*	1.07	(1.00, 1.15)		1.44	(0.99, 2.07)	
	*60–74*	0.80	(0.75, 0.84)		0.98	(0.70, 1.36)	
	*75–89*	0.64	(0.61, 0.67)		0.57	(0.43, 0.75)	
	*90–104*	0.54	(0.51, 0.57)		0.35	(0.24, 0.50)	
	*105* +	0.45	(0.41, 0.49)		0.13	(0.04, 0.41)	
Male	< *30*	2.96	(2.37, 3.69)		8.61	(4.32, 17.19)	
	*30–44*	1.96	(1.73, 2.23)		3.97	(2.43, 6.51)	
	*45–59*	1.53	(1.43, 1.64)		2.55	(1.83, 3.55)	
	*60–74*	1.35	(1.28, 1.42)		1.95	(1.46, 2.59)	
	*75–89*	1.12	(1.08, 1.17)		1.10	(0.86, 1.41)	
	*90–104*	ref	-		ref	-	
	*105* +	0.92	(0.87, 0.98)		0.84	(0.55, 1.28)	
**eGFRCr-Cys**				< 0.001			0.1
Female	< *30*	2.15	(1.59, 2.90)		6.74	(2.74, 16.57)	
	*30–44*	1.41	(1.17, 1.70)		1.37	(0.51, 3.73)	
	*45–59*	1.01	(0.93, 1.10)		1.20	(0.75, 1.91)	
	*60–74*	0.80	(0.75, 0.85)		0.90	(0.63, 1.30)	
	*75–89*	0.65	(0.62, 0.67)		0.64	(0.50, 0.81)	
	*90–104*	0.55	(0.53, 0.57)		0.36	(0.26, 0.49)	
	*105* +	0.54	(0.50, 0.58)		0.34	(0.18, 0.65)	
Male	< *30*	2.58	(2.01, 3.30)		4.90	(2.00, 12.04)	
	*30–44*	1.93	(1.65, 2.27)		4.02	(2.26, 7.17)	
	*45–59*	1.48	(1.36, 1.61)		2.16	(1.44, 3.24)	
	*60–74*	1.25	(1.18, 1.33)		2.14	(1.60, 2.85)	
	*75–89*	1.11	(1.08, 1.15)		1.11	(0.90, 1.38)	
	*90–104*	ref	-		ref	-	
	*105* +	0.94	(0.88, 1.00)		0.91	(0.58, 1.43)	
(B) Heart failure							
**eGFRCr**				0.1			0.3
Female	< *30*	3.33	(2.07, 5.38)		4.35	(1.39, 13.64)	
	*30–44*	1.80	(1.31, 2.46)		2.02	(0.89, 4.56)	
	*45–59*	0.96	(0.80, 1.14)		0.76	(0.44, 1.31)	
	*60–74*	0.79	(0.69, 0.89)		0.88	(0.62, 1.26)	
	*75–89*	0.65	(0.60, 0.70)		0.49	(0.38, 0.62)	
	*90–104*	0.61	(0.57, 0.65)		0.47	(0.37, 0.59)	
	*105* +	0.92	(0.79, 1.08)		1.05	(0.66, 1.67)	
Male	< *30*	5.22	(3.78, 7.21)		2.63	(0.84, 8.26)	
	*30–44*	2.58	(1.99, 3.35)		2.76	(1.41, 5.40)	
	*45–59*	1.52	(1.30, 1.77)		2.11	(1.46, 3.06)	
	*60–74*	1.16	(1.04, 1.30)		1.20	(0.87, 1.67)	
	*75–89*	0.91	(0.85, 0.98)		0.95	(0.79, 1.16)	
	*90–104*	ref	-		ref	-	
	*105* +	1.55	(1.37, 1.75)		1.98	(1.41, 2.79)	
**eGFRCys**				< 0.001			0.05
Female	< *30*	3.88	(2.64, 5.69)		7.95	(3.66, 17.27)	
	*30–44*	3.01	(2.48, 3.66)		3.93	(2.38, 6.50)	
	*45–59*	1.89	(1.68, 2.12)		1.99	(1.41, 2.81)	
	*60–74*	1.16	(1.04, 1.29)		1.11	(0.80, 1.55)	
	*75–89*	0.76	(0.69, 0.83)		0.62	(0.46, 0.82)	
	*90–104*	0.52	(0.47, 0.57)		0.30	(0.20, 0.44)	
	*105* +	0.39	(0.32, 0.48)		0.32	(0.15, 0.66)	
Male	< *30*	7.16	(5.43, 9.44)		8.96	(4.49, 17.91)	
	*30–44*	3.77	(3.14, 4.52)		6.14	(3.99, 9.45)	
	*45–59*	2.16	(1.91, 2.43)		2.84	(2.04, 3.95)	
	*60–74*	1.83	(1.65, 2.01)		2.45	(1.85, 3.24)	
	*75–89*	1.24	(1.15, 1.34)		1.33	(1.04, 1.70)	
	*90–104*	ref	-		ref	-	
	*105* +	0.82	(0.72, 0.93)		0.64	(0.40, 1.01)	
**eGFRCr-Cys**				< 0.001			0.03
Female	< *30*	4.19	(2.79, 6.28)		8.76	(3.83, 20.02)	
	*30–44*	2.51	(1.92, 3.28)		1.47	(0.54, 4.00)	
	*45–59*	1.68	(1.46, 1.93)		2.47	(1.70, 3.58)	
	*60–74*	1.15	(1.03, 1.29)		1.02	(0.70, 1.47)	
	*75–89*	0.78	(0.72, 0.84)		0.74	(0.58, 0.95)	
	*90–104*	0.53	(0.48, 0.58)		0.34	(0.24, 0.48)	
	*105* +	0.62	(0.53, 0.72)		0.66	(0.40, 1.08)	
Male	< *30*	6.26	(4.64, 8.44)		6.33	(2.78, 14.45)	
	*30–44*	3.71	(2.99, 4.60)		4.98	(2.89, 8.59)	
	*45–59*	2.11	(1.83, 2.44)		3.13	(2.17, 4.51)	
	*60–74*	1.56	(1.40, 1.74)		2.14	(1.58, 2.89)	
	*75–89*	1.18	(1.10, 1.27)		1.45	(1.17, 1.79)	
	*90–104*	ref	-		ref	-	
	*105* +	0.95	(0.83, 1.09)		1.16	(0.77, 1.74)	
**eGFRCr**				0.09			0.07
Female	< *30*	1.74	(1.19, 2.54)		4.89	(2.61, 9.16)	
	*30–44*	1.16	(0.92, 1.46)		1.77	(1.06, 2.96)	
	*45–59*	0.75	(0.67, 0.83)		0.66	(0.47, 0.93)	
	*60–74*	0.63	(0.58, 0.68)		0.39	(0.29, 0.52)	
	*75–89*	0.60	(0.58, 0.63)		0.31	(0.27, 0.36)	
	*90–104*	0.59	(0.57, 0.61)		0.34	(0.29, 0.39)	
	*105* +	0.58	(0.54, 0.64)		0.55	(0.41, 0.75)	
Male	< *30*	3.21	(2.49, 4.14)		5.04	(3.06, 8.29)	
	*30–44*	1.35	(1.10, 1.65)		2.74	(1.86, 4.03)	
	*45–59*	1.24	(1.13, 1.37)		1.56	(1.23, 1.98)	
	*60–74*	1.07	(1.00, 1.14)		1.06	(0.88, 1.28)	
	*75–89*	1.02	(0.98, 1.05)		0.97	(0.87, 1.07)	
	*90–104*	ref	-		ref	-	
	*105* +	1.11	(1.04, 1.18)		1.76	(1.47, 2.09)	
**eGFRCys**				< 0.001			< 0.001
Female	< *30*	1.75	(1.29, 2.36)		5.41	(3.32, 8.84)	
	*30–44*	1.11	(0.95, 1.30)		1.91	(1.35, 2.72)	
	*45–59*	0.96	(0.89, 1.03)		0.85	(0.67, 1.07)	
	*60–74*	0.78	(0.73, 0.83)		0.51	(0.41, 0.63)	
	*75–89*	0.64	(0.61, 0.67)		0.37	(0.31, 0.43)	
	*90–104*	0.54	(0.52, 0.57)		0.24	(0.20, 0.30)	
	*105* +	0.43	(0.39, 0.48)		0.25	(0.16, 0.38)	
Male	< *30*	2.96	(2.37, 3.69)		7.29	(4.92, 10.82)	
	*30–44*	1.57	(1.37, 1.80)		2.96	(2.23, 3.94)	
	*45–59*	1.34	(1.25, 1.44)		1.97	(1.65, 2.37)	
	*60–74*	1.25	(1.18, 1.32)		1.53	(1.32, 1.79)	
	*75–89*	1.12	(1.08, 1.16)		1.15	(1.02, 1.30)	
	*90–104*	ref	-		ref	-	
	*105* +	0.94	(0.88, 0.99)		0.81	(0.66, 0.99)	
**eGFRCr-Cys**				< 0.001			0.003
Female	< *30*	1.77	(1.26, 2.49)		4.42	(2.42, 8.06)	
	*30–44*	1.27	(1.03, 1.56)		2.44	(1.60, 3.72)	
	*45–59*	0.91	(0.82, 1.00)		0.85	(0.63, 1.14)	
	*60–74*	0.79	(0.73, 0.84)		0.51	(0.40, 0.66)	
	*75–89*	0.64	(0.62, 0.67)		0.39	(0.34, 0.45)	
	*90–104*	0.56	(0.53, 0.58)		0.28	(0.23, 0.34)	
	*105* +	0.48	(0.44, 0.52)		0.35	(0.26, 0.49)	
Male	< *30*	2.92	(2.29, 3.71)		6.20	(4.00, 9.63)	
	*30–44*	1.73	(1.46, 2.05)		3.00	(2.12, 4.25)	
	*45–59*	1.25	(1.14, 1.37)		1.98	(1.59, 2.47)	
	*60–74*	1.21	(1.14, 1.29)		1.55	(1.32, 1.83)	
	*75–89*	1.10	(1.06, 1.14)		1.14	(1.02, 1.27)	
	*90–104*	ref	-		ref	-	
	*105* +	0.94	(0.89, 1.00)		1.07	(0.88, 1.30)	

eGFR is measured in mL/min/1.73m^2^. *P*-values are for the interaction between eGFR and sex

### All-cause cardiac deaths

In adjusted models for any cardiac death, sex was a significant effect modifier with eGFRCys and eGFRCr-Cys, but not eGFRCr (interaction term for sex with: eGFRCr = *p* = 0.06; eGFRCys = *p* < 0.001; eGFRCr-Cys = *p* < 0.001). Unadjusted model estimates are given in Additional File 1: Table S4 and adjusted model estimates are given in Additional File 1: Table S5.

**Fig. 4:**
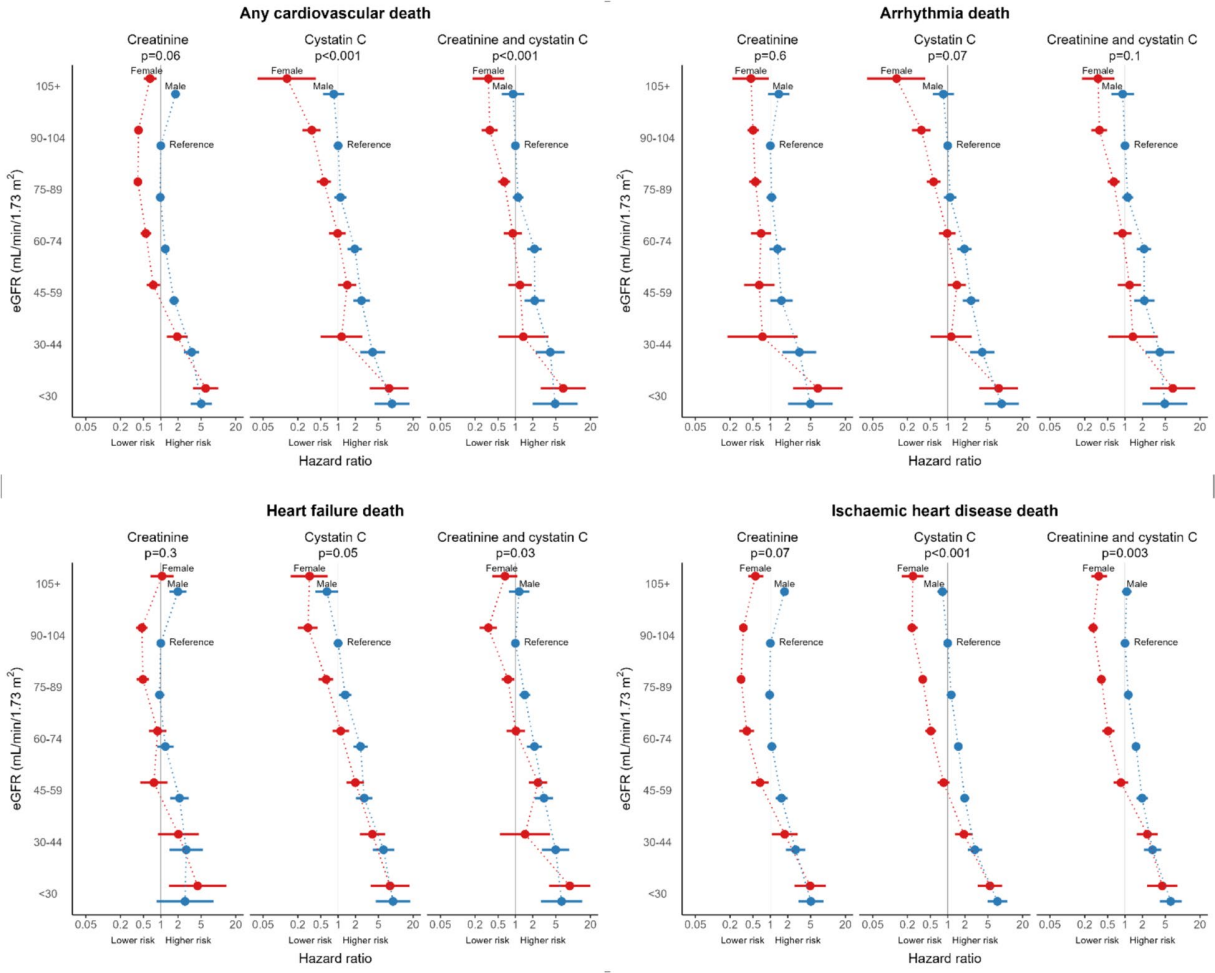
Forest plots of time to any cardiac death and cardiac subtype death by eGFR and sex. eGFR is measured in mL/min/1.73m^2^. P-values are for the interaction between eGFR and sex

The risk of cardiac death was increased in both sexes for lower eGFRCr, eGFRCys and eGFRCr-Cys, more so among males (Fig. 4, Table 3). Males had over eight times the risk (HR 8.61, 95% CI 4.32, 17.19), and females had over seven times the risk of cardiac death (HR 7.66, 95% CI 3.53, 16.61) when eGFRCys < 30 mL/min/1.73m^2^ compared to males with eGFRCys 90–104 mL/min/1.73m^2^ in adjusted models.

In models with eGFRCr only, the risk of cardiac death was increased in males with eGFRCr ≥ 105 mL/min/1.73m^2^ (HR 1.80, 95% CI 1.55, 2.11) compared to males with eGFRCys 90–104 mL/min/1.73m^2^. In models with eGFRCys or eGFRCr-Cys, the risk of cardiac death had a more linear relationship with increasing eGFR. Both sexes had a trend of lower risk as eGFR increased to ≥ 105 mL/min/1.73m^2^ (eGFRCys: females HR 0.13, 95% CI 0.04, 0.41; males HR 0.84, 95% CI 0.55, 1.28; eGFRCr-Cys: females HR 0.34, 95% CI 0.18, 0.65; males HR 0.91, 95% CI 0.58, 1.43) compared to males with eGFR 90–104 mL/min/1.73m^2^. However, cardiac death risk in males with eGFR ≥ 105 mL/min/1.73m^2^ was not significantly different from males with eGFR 90–104 mL/min/1.73m^2^.

Model performance was similar between all eGFR models, with eGFRCys having slightly lower AIC (Additional File 1: Table S3). The calibration plot did not depict any substantial major deviations from the line of equality between the adjusted models. However, eGFR-Cys and eGFR-CrCys did maintain closer proximity to the line of equality, particularly when the occurrence of deaths reduced (Additional File 2: Figure S1).

The three-way interaction term with age < 50 years compared to age ≥ 50 years was unable to properly converge in any of the eGFRCr, eGFRCys or eGFRCrCys due to the reduced number of deaths per stratum. The two-way interaction term with eGFRCys/eGFRCr ratio and sex was significant in the association with mortality (*p* < 0.001). The mortality risk for females with eGFRCys/eGFRCr < 1 was 2 × higher than females with eGFRCys/eGFRCr ≥ 1 (HR 2.01, 95% CI 1.73, 2.34), while males with eGFRCys/eGFRCr < 1 only had a 40% increased mortality risk compared to males with eGFRCys/eGFRCr ≥ 1 (HR 1.40, 95% CI 1.29, 1.53).

There were differences by cardiac death subtype (Table 4, Fig. 3, Additional File 1: Table S4, Additional File 1: Table S5). Sex and eGFRCr were not effect modifiers for any cardiac death subtype. Sex and eGFRCys or eGFRCr-Cys were effect modifiers for heart failure and ischaemic heart disease deaths only. Like the overall results, when using eGFRCys or eGFRCr-Cys, the risk of death from heart failure or ischaemic heart disease in those with ≥ 105 mL/min/1.73m^2^ was reduced in females and not significantly different in males compared to males with 90–104 mL/min/1.73m^2^. Females had an increased risk of heart failure death when eGFRCys was 45–59 mL/min/1.73m^2^ (HR 1.99, 95% CI 1.41, 2.81) compared to males with eGFRCys 90–104 mL/min/1.73m^2^, but an increase in risk was not detected at the same level of eGFRCr.

## Discussion

In this study, we have demonstrated that there are sex differences which modified the association between cardiac risk and kidney function, and this relationship varies by biomarker used to estimate kidney function. When used to estimate kidney function, cystatin C reclassified approximately a third of people to lower ranges of eGFR compared to creatinine in both females and males. The risk of cardiac events and deaths was increased at lower ranges of eGFRCr, eGFRCys and eGFRCr-Cys in both sexes, but more so in males. However, the risk of cardiac events and deaths was also increased at higher ranges of eGFRCr in males, but not females. Using eGFRCys both females and males had lower risk of cardiac events and deaths with higher eGFR. Similar findings were reflected across cardiac subtypes. It was possible to detect an increased risk of cardiac events in females with eGFR < 60 mL/min/1.73m^2^ when using eGFRCys, but not eGFRCr. This sex difference was exacerbated in heart failure events and death. We also found the risk of cardiac events and deaths was increased in females with eGFRCys/eGFRCr ratio below one, compared to their male counterparts.

Our findings unveiled sex differences seldom described in the published literature on cystatin C and its association with cardiac outcomes. Consistent with other literature, lower eGFRCys was more strongly and linearly associated with increased risk of cardiac event and death outcomes compared to eGFRCr [24–27]. Males had consistently higher risk of cardiac events and death at the same eGFR compared to females, as reported in other studies [28, 29]. While prior studies have reported sex-stratified cardiovascular risk by eGFR and discordance between eGFRCr and eGFRCys [30], these have not fully interrogated differences when using eGFRCys compared to eGFRCr, nor sex as a modifying factor [26, 31]. We found this association was modified by sex, indicating the trend in cardiac risk across different eGFRCys was fundamentally different for females compared to males. Females had increased cardiac risk at higher eGFRCys categories than when using eGFRCr. This suggests females may be overlooked as a high-risk group for cardiac events and death when based on eGFRCr.

Sex differences in risk factors and outcomes of cardiovascular disease have been historically under-appreciated: growing evidence suggests females with cardiovascular disease have comparatively poorer outcomes than their male counterparts [32, 33]. Previous studies have shown inclusion of eGFRCys improves risk discrimination for cardiovascular outcomes in risk calculators [25]. Our work has further added that eGFRCys has greater added value in females. While the mechanisms are uncertain, it is plausible that cardiac risk associated with lower eGFR is more sensitively detected by eGFRCys in females, owing to their muscle mass, lower decrease in muscle mass with older age and lower burden of cardiometabolic disease [34–36]. Larger differences between eGFRCys and eGFRCr in females were also observed in our study and were associated with greater risk of cardiac events and deaths. Shrunken pore syndrome, a selective glomerular hypofiltration syndrome, has been described as a potential mechanism for this whereby there is a shrinking or elongation of the pores for glomerular filtration [27, 37]. An alternative explanation is differential balance of non-GFR determinants of creatinine and cystatin C in male and female participants. Other studies have shown abnormally low eGFRCys/eGFRCr is more common in females and is strongly associated with mortality, morbidity, heart failure and kidney failure [38–41]. Our study demonstrated a sex difference in cardiac outcomes as well, where females with eGFRCys/eGFRCr < 1 had higher risk of cardiac events and deaths compared to their male counterparts. We were unable to further explore lower ranges of eGFRCys/eGFRCr due to fewer observations with eGFRCys/eGFRCr below 0.8. Other sex-based confounders related to cystatin C are less well understood compared to creatinine, but these may include corticosteroid therapy and thyroid dysfunction [42, 43].

Supporting the veracity of our findings, the results we report for eGFRCys have been seen similarly with cardiac troponins. Cardiac troponins are typically lower in females, rise more steeply with age in females, and are predictive of important adverse cardiovascular outcomes at lower thresholds in females compared to males [44, 45]. Consideration is now being given to sex-specific thresholds in cardiac troponins for identifying risk. Our data suggested that similar strategies may be warranted for eGFRCys.

Importantly, the improvements in risk detection using eGFRCys, particularly in females, are most pronounced with mild reductions in eGFR, when patients are likely to be managed in primary care and when primary prevention strategies (including statins and SGLT2 inhibitors) are most likely to be effective. As shown previously [46], the biologically implausible relationship between higher eGFR and higher cardiac risk is not observed for eGFRCys, as it is for eGFRCr, supporting eGFRCys as a more robust predictor for cardiac risk for both sexes than eGFRCr.

Our study is strengthened by using a large sample of people from the general population, including rich patient data, both biochemical and medical events, over a substantial follow-up. However, our study did have limitations. First, the UK Biobank is limited to those aged 40–73 years, and our findings may not generalise to younger age groups. Second, eGFR was based on a single measure collected at baseline, where most had eGFR > 60 mL/min/1.73m^2^ (98% eGFRCr; 96% eGFRCys). While cystatin C assays were standardised only after 2010, all biochemical data were processed in a centralised laboratory in accordance with published protocols [47]. This would result in consistent and reliable measurement of creatinine and cystatin C across all participants. We were unable to account for changes in kidney function over the follow-up period; regardless, a single measurement at baseline is a reasonable estimate [14]. Third, we used the CKD-EPI 2009 equation for the eGFR calculations, and variations with other eGFR equations do exist. However, these eGFR equations have not been validated in UK populations, and the CKD-EPI 2009 remains the recommended equation at the time of our study and currently for UK populations as per NICE guidelines [48]. Further, it is important to note that there is uncertainty at higher ranges of eGFR values (e.g. above ~ 100 mL/min/1.73m^2^), where very high values may reflect falsely inflated estimates in non-standard physiological states. Fourth, we did not additionally adjust for the non-GFR determinants of either creatinine or cystatin C (e.g. corticosteroid use), as these factors are not currently considered in most cardiovascular risk prediction tools. Additional adjustment for these non-GFR determinants, which also show differences by sex, could plausibly alter the sex differences in reported risk associations, though this is expected to be minimal. Fifth, we have inadequate data to comment on the cost effectiveness of cystatin C testing for cardiovascular risk stratification in a setting where cystatin C may be more expensive and less readily available than serum creatinine. However, the additional cost of cystatin C alongside serum creatinine where cystatin C use is widespread (such as in Sweden) and the added health benefits likely outweigh these costs [49].

## Conclusions

In conclusion, we have shown that evaluating kidney function using serum creatinine may not identify increased cardiac risk among females as effectively as cystatin C. Incorporating cystatin C into eGFR provides a different estimate than serum creatinine alone, which may be particularly relevant in females. Further, cystatin C may provide improved cardiovascular risk stratification, particularly in females. Increased cardiac risk was detected in females when eGFRCys mL/min/1.73 m^2^, but not until eGFRCr was < 45 mL/min/1.73 m^2^. Incorporating eGFRCys, rather than eGFRCr, into cardiovascular risk assessment may be more important for early detection among high-risk females with CKD.

## Supplementary Information

Additional file 1. Table S1–S5. Table S1: Unadjusted estimates of analysis of time to first cardiac event. Table S2: Adjusted estimates of analysis of time to first cardiac event. Table S3: Comparison of AIC and C-statistic for adjusted models. Table S4: Unadjusted estimates of analysis of time to cardiac death. Table S5: Adjusted estimates of analysis of time to cardiac death

Additional file 2. Figure S1: Calibration plots for adjusted models for analysis of time to: (A) first cardiac event and (B) first cardiac death

## Data Availability

The UK Biobank data underlying this article are available from the UK Biobank (www.ukbiobank.ac.uk). The study application ID is 69,891.

## Abbreviations

AIC: Akaike Information Criterion
CKD: Chronic kidney disease
Cr: Serum creatinine
Cys: Cystatin C
eGFR: Estimated glomerular filtration rate
HDL cholesterol: High-density lipoprotein cholesterol
HR: Hazard ratio
ICD: International Classification of Diseases and Related Health Problems
IQI: Interquartile interval
