# Supplementary material for: Sex differences in cardiac risk and kidney function: serum creatinine versus cystatin C
Source: BMC Med. 2025 Dec 30;24:62. doi: 10.1186/s12916-025-04588-9 (PMC12860083; doi:10.1186/s12916-025-04588-9)
Supplement: Supplementary file 1 — Additional file 1. Table S1–S5. Table S1: Unadjusted estimates of analysis of time to first cardiac event. Table S2: Adjusted estimates of analysis of time to first cardiac event. Table S3: Comparison of AIC and C-statistic for adjusted models. Table S4: Unadjusted estimates of analysis of time to cardiac death. Table S5: Adjusted estimates of analysis of time to cardiac death [file 12916_2025_4588_MOESM1_ESM.docx]

Table S1. Unadjusted estimates of analysis of time to first cardiac event.

|  | **Arrythmia** | | | **Heart failure** | | | **Ischaemic heart disease** | | | **Any cardiac event** | | |
| --- | --- | --- | --- | --- | --- | --- | --- | --- | --- | --- | --- | --- |
| **Characteristic** | *HR* | *(95% CI)* | *p* | *HR* | *(95% CI)* | *p* | *HR* | *(95% CI)* | *p* | *HR* | *(95% CI)* | *p* |
| **eGFRCr and sex** |  |  | 0.02 |  |  | 0.1 |  |  | <0.001 |  |  | <0.001 |
| Female |  |  |  |  |  |  |  |  |  |  |  |  |
| *<30* | 3.72 | (2.72, 5.10) |  | 7.95 | (5.27, 11.99) |  | 3.14 | (2.23, 4.41) |  | 3.86 | (3.01, 4.96) |  |
| *30-44* | 2.46 | (2.03, 2.96) |  | 3.85 | (2.87, 5.16) |  | 1.85 | (1.49, 2.30) |  | 2.16 | (1.85, 2.53) |  |
| *45-59* | 1.31 | (1.20, 1.43) |  | 1.57 | (1.34, 1.85) |  | 1.02 | (0.92, 1.14) |  | 1.21 | (1.13, 1.30) |  |
| *60-74* | 0.91 | (0.85, 0.97) |  | 1.08 | (0.96, 1.22) |  | 0.73 | (0.67, 0.78) |  | 0.85 | (0.81, 0.90) |  |
| *75-89* | 0.69 | (0.66, 0.71) |  | 0.69 | (0.65, 0.74) |  | 0.56 | (0.53, 0.58) |  | 0.65 | (0.63, 0.67) |  |
| *90-104* | 0.56 | (0.54, 0.58) |  | 0.53 | (0.50, 0.57) |  | 0.47 | (0.46, 0.49) |  | 0.54 | (0.53, 0.56) |  |
| *105+* | 0.24 | (0.23, 0.26) |  | 0.26 | (0.23, 0.30) |  | 0.23 | (0.22, 0.25) |  | 0.26 | (0.24, 0.27) |  |
| Male |  |  |  |  |  |  |  |  |  |  |  |  |
| *<30* | 4.79 | (3.74, 6.14) |  | 11.70 | (8.59, 15.94) |  | 5.70 | (4.46, 7.27) |  | 5.03 | (4.10, 6.17) |  |
| *30-44* | 3.34 | (2.80, 3.99) |  | 6.70 | (5.26, 8.55) |  | 2.85 | (2.35, 3.46) |  | 3.31 | (2.87, 3.82) |  |
| *45-59* | 2.30 | (2.11, 2.51) |  | 3.20 | (2.77, 3.70) |  | 2.17 | (1.98, 2.38) |  | 2.23 | (2.08, 2.39) |  |
| *60-74* | 1.68 | (1.58, 1.78) |  | 1.95 | (1.74, 2.17) |  | 1.58 | (1.49, 1.68) |  | 1.62 | (1.55, 1.70) |  |
| *75-89* | 1.28 | (1.24, 1.32) |  | 1.21 | (1.14, 1.29) |  | 1.23 | (1.19, 1.27) |  | 1.24 | (1.21, 1.27) |  |
| *90-104* | ref | - |  | ref | - |  | ref | - |  | ref | - |  |
| *105+* | 0.49 | (0.46, 0.52) |  | 0.60 | (0.54, 0.67) |  | 0.64 | (0.60, 0.67) |  | 0.58 | (0.55, 0.60) |  |
| **eGFRCr** |  |  | <0.001 |  |  | <0.001 |  |  | <0.001 |  |  | <0.001 |
| *<30* | 5.74 | (4.72, 6.97) |  | 13.54 | (10.57, 17.33) |  | 6.37 | (5.22, 7.77) |  | 6.06 | (5.17, 7.10) |  |
| *30-44* | 3.80 | (3.34, 4.32) |  | 6.97 | (5.78, 8.40) |  | 3.28 | (2.84, 3.80) |  | 3.61 | (3.25, 4.02) |  |
| *45-59* | 2.25 | (2.11, 2.40) |  | 2.99 | (2.68, 3.34) |  | 2.09 | (1.95, 2.24) |  | 2.16 | (2.05, 2.27) |  |
| *60-74* | 1.62 | (1.54, 1.69) |  | 1.94 | (1.79, 2.10) |  | 1.52 | (1.45, 1.60) |  | 1.56 | (1.51, 1.62) |  |
| *75-89* | 1.26 | (1.23, 1.29) |  | 1.26 | (1.20, 1.32) |  | 1.22 | (1.19, 1.25) |  | 1.23 | (1.21, 1.25) |  |
| *90-104* | ref | - |  | ref | - |  | ref | - |  | ref | - |  |
| *105+* | 0.46 | (0.44, 0.49) |  | 0.55 | (0.51, 0.60) |  | 0.58 | (0.55, 0.61) |  | 0.53 | (0.51, 0.55) |  |
| **eGFRCys and sex** |  |  | <0.001 |  |  | <0.001 |  |  | <0.001 |  |  | <0.001 |
| Female |  |  |  |  |  |  |  |  |  |  |  |  |
| *<30* | 4.51 | (3.54, 5.76) |  | 10.05 | (7.17, 14.08) |  | 3.43 | (2.60, 4.52) |  | 4.48 | (3.68, 5.45) |  |
| *30-44* | 2.94 | (2.59, 3.35) |  | 7.08 | (5.91, 8.49) |  | 2.23 | (1.93, 2.59) |  | 2.81 | (2.53, 3.11) |  |
| *45-59* | 2.01 | (1.88, 2.14) |  | 3.72 | (3.34, 4.14) |  | 1.61 | (1.50, 1.72) |  | 1.90 | (1.81, 2.00) |  |
| *60-74* | 1.34 | (1.27, 1.41) |  | 1.90 | (1.71, 2.10) |  | 1.11 | (1.04, 1.17) |  | 1.26 | (1.21, 1.31) |  |
| *75-89* | 0.87 | (0.83, 0.90) |  | 0.94 | (0.87, 1.03) |  | 0.70 | (0.67, 0.73) |  | 0.82 | (0.80, 0.85) |  |
| *90-104* | 0.53 | (0.51, 0.56) |  | 0.47 | (0.42, 0.51) |  | 0.43 | (0.41, 0.46) |  | 0.51 | (0.49, 0.52) |  |
| *105+* | 0.22 | (0.21, 0.24) |  | 0.16 | (0.14, 0.20) |  | 0.19 | (0.17, 0.21) |  | 0.22 | (0.21, 0.24) |  |
| Male |  |  |  |  |  |  |  |  |  |  |  |  |
| *<30* | 6.01 | (4.90, 7.37) |  | 16.45 | (12.70, 21.30) |  | 6.10 | (4.95, 7.52) |  | 6.36 | (5.39, 7.51) |  |
| *30-44* | 4.42 | (3.92, 4.98) |  | 10.31 | (8.70, 12.22) |  | 3.77 | (3.31, 4.29) |  | 4.20 | (3.81, 4.64) |  |
| *45-59* | 3.11 | (2.92, 3.31) |  | 4.95 | (4.43, 5.54) |  | 2.76 | (2.58, 2.96) |  | 2.99 | (2.84, 3.14) |  |
| *60-74* | 2.39 | (2.27, 2.50) |  | 3.45 | (3.15, 3.78) |  | 2.18 | (2.07, 2.29) |  | 2.30 | (2.21, 2.39) |  |
| *75-89* | 1.52 | (1.46, 1.57) |  | 1.70 | (1.58, 1.84) |  | 1.48 | (1.43, 1.54) |  | 1.49 | (1.45, 1.53) |  |
| *90-104* | ref | - |  | ref | - |  | ref | - |  | ref | - |  |
| *105+* | 0.64 | (0.60, 0.68) |  | 0.56 | (0.50, 0.64) |  | 0.69 | (0.65, 0.73) |  | 0.68 | (0.65, 0.71) |  |
| **eGFRCys** |  |  | <0.001 |  |  | <0.001 |  |  | <0.001 |  |  | <0.001 |
| *<30* | 7.34 | (6.28, 8.58) |  | 19.59 | (15.94, 24.07) |  | 7.21 | (6.10, 8.52) |  | 7.71 | (6.80, 8.76) |  |
| *30-44* | 4.98 | (4.56, 5.44) |  | 12.50 | (11.03, 14.17) |  | 4.40 | (3.99, 4.86) |  | 4.86 | (4.52, 5.21) |  |
| *45-59* | 3.39 | (3.24, 3.55) |  | 6.19 | (5.71, 6.70) |  | 3.13 | (2.98, 3.29) |  | 3.31 | (3.19, 3.43) |  |
| *60-74* | 2.47 | (2.38, 2.56) |  | 3.77 | (3.51, 4.04) |  | 2.37 | (2.28, 2.46) |  | 2.41 | (2.34, 2.48) |  |
| *75-89* | 1.62 | (1.57, 1.66) |  | 1.91 | (1.80, 2.02) |  | 1.60 | (1.56, 1.65) |  | 1.60 | (1.57, 1.64) |  |
| *90-104* | ref | - |  | ref | - |  | ref | - |  | ref | - |  |
| *105+* | 0.58 | (0.55, 0.60) |  | 0.51 | (0.46, 0.57) |  | 0.64 | (0.61, 0.67) |  | 0.61 | (0.59, 0.64) |  |
| **eGFRCr-Cys and sex** |  |  | 0.006 |  |  | 0.001 |  |  | <0.001 |  |  | <0.001 |
| Female |  |  |  |  |  |  |  |  |  |  |  |  |
| *<30* | 4.72 | (3.59, 6.20) |  | 10.84 | (7.55, 15.57) |  | 3.49 | (2.56, 4.76) |  | 4.64 | (3.73, 5.79) |  |
| *30-44* | 3.08 | (2.59, 3.67) |  | 6.40 | (5.00, 8.20) |  | 2.44 | (2.01, 2.96) |  | 2.86 | (2.48, 3.29) |  |
| *45-59* | 2.03 | (1.87, 2.20) |  | 3.55 | (3.11, 4.05) |  | 1.57 | (1.43, 1.72) |  | 1.91 | (1.78, 2.04) |  |
| *60-74* | 1.39 | (1.31, 1.48) |  | 1.99 | (1.79, 2.21) |  | 1.13 | (1.06, 1.20) |  | 1.30 | (1.24, 1.37) |  |
| *75-89* | 0.90 | (0.87, 0.93) |  | 1.01 | (0.94, 1.09) |  | 0.71 | (0.68, 0.74) |  | 0.84 | (0.82, 0.86) |  |
| *90-104* | 0.54 | (0.52, 0.56) |  | 0.47 | (0.43, 0.51) |  | 0.44 | (0.42, 0.46) |  | 0.51 | (0.50, 0.53) |  |
| *105+* | 0.29 | (0.27, 0.31) |  | 0.28 | (0.24, 0.33) |  | 0.24 | (0.22, 0.26) |  | 0.28 | (0.27, 0.30) |  |
| Male |  |  |  |  |  |  |  |  |  |  |  |  |
| *<30* | 5.60 | (4.46, 7.03) |  | 15.52 | (11.71, 20.58) |  | 6.10 | (4.86, 7.67) |  | 5.89 | (4.89, 7.09) |  |
| *30-44* | 4.47 | (3.84, 5.20) |  | 10.55 | (8.61, 12.93) |  | 3.96 | (3.38, 4.65) |  | 4.32 | (3.82, 4.88) |  |
| *45-59* | 3.31 | (3.05, 3.60) |  | 5.41 | (4.74, 6.19) |  | 2.70 | (2.47, 2.95) |  | 3.04 | (2.84, 3.25) |  |
| *60-74* | 2.40 | (2.27, 2.53) |  | 3.28 | (2.97, 3.63) |  | 2.19 | (2.07, 2.32) |  | 2.32 | (2.22, 2.42) |  |
| *75-89* | 1.62 | (1.57, 1.67) |  | 1.79 | (1.67, 1.91) |  | 1.51 | (1.46, 1.56) |  | 1.55 | (1.51, 1.59) |  |
| *90-104* | ref | - |  | ref | - |  | ref | - |  | ref | - |  |
| *105+* | 0.56 | (0.53, 0.60) |  | 0.57 | (0.50, 0.64) |  | 0.64 | (0.60, 0.68) |  | 0.62 | (0.59, 0.65) |  |
| **eGFRCr-Cys** |  |  | <0.001 |  |  | <0.001 |  |  | <0.001 |  |  | <0.001 |
| *<30* | 7.01 | (5.88, 8.35) |  | 18.93 | (15.14, 23.66) |  | 7.05 | (5.87, 8.48) |  | 7.30 | (6.33, 8.42) |  |
| *30-44* | 5.05 | (4.50, 5.66) |  | 11.89 | (10.15, 13.92) |  | 4.61 | (4.07, 5.22) |  | 4.88 | (4.45, 5.35) |  |
| *45-59* | 3.40 | (3.21, 3.61) |  | 6.07 | (5.51, 6.68) |  | 2.92 | (2.74, 3.12) |  | 3.22 | (3.07, 3.38) |  |
| *60-74* | 2.43 | (2.33, 2.53) |  | 3.57 | (3.31, 3.86) |  | 2.27 | (2.17, 2.37) |  | 2.36 | (2.28, 2.43) |  |
| *75-89* | 1.65 | (1.61, 1.69) |  | 1.93 | (1.83, 2.03) |  | 1.56 | (1.52, 1.60) |  | 1.59 | (1.56, 1.62) |  |
| *90-104* | ref | - |  | ref | - |  | ref | - |  | ref | - |  |
| *105+* | 0.54 | (0.52, 0.57) |  | 0.56 | (0.51, 0.62) |  | 0.58 | (0.56, 0.61) |  | 0.58 | (0.56, 0.60) |  |
| **Female** | 0.55 | (0.54, 0.56) | <0.001 | 0.54 | (0.52, 0.57) | <0.001 | 0.46 | (0.45, 0.47) | <0.001 | 0.53 | (0.52, 0.54) | <0.001 |
| **Age (per year)** | 1.10 | (1.10, 1.10) | <0.001 | 1.11 | (1.11,1.12) | <0.001 | 1.07 | (1.07, 1.07) | <0.001 | 1.08 | (1.08, 1.09) | <0.001 |
| **Smoker** |  |  | <0.001 |  |  | <0.001 |  |  | <0.001 |  |  | <0.001 |
| Never | ref | - |  | ref | - |  | ref | - |  | ref | - |  |
| Previous | 1.44 | (1.40, 1.47) |  | 1.56 | (1.48, 1.63) |  | 1.45 | (1.41, 1.49) |  | 1.42 | (1.39, 1.45) |  |
| Current | 1.24 | (1.19, 1.28) |  | 2.05 | (1.93, 2.18) |  | 1.75 | (1.69, 1.82) |  | 1.49 | (1.45, 1.53) |  |
| **Comorbidities** |  |  |  |  |  |  |  |  |  |  |  |  |
| Diabetes | 2.11 | (2.03, 2.20) | <0.001 | 3.58 | (3.37, 3.81) | <0.001 | 2.72 | (2.62, 2.83) | <0.001 | 2.33 | (2.26, 2.40) | <0.001 |
| Cancer | 1.45 | (1.40, 1.50) | <0.001 | 1.72 | (1.61, 1.84) | <0.001 | 2.42 | (1.22, 1.33) | <0.001 | 1.40 | (1.36, 1.44) | <0.001 |
| Hypertension | 2.19 | (2.14, 2.23) | <0.001 | 2.81 | (2.69, 2.93) | <0.001 | 2.26 | (2.21, 2.32) | <0.001 | 2.17 | (2.13, 2.21) | <0.001 |
| Hyperlipidaemia | 2.10 | (2.05, 2.16) | <0.001 | 2.72 | (2.59, 2.85) | <0.001 | 2.41 | (2.34, 2.47) | <0.001 | 2.21 | (2.17, 2.26) | <0.001 |
| **Total:HDL cholesterol** | 1.04 | (1.03, 1.05) | <0.001 | 1.10 | (1.07, 1.12) | <0.001 | 1.30 | (1.28, 1.31) | <0.001 | 1.15 | (1.15, 1.16) | <0.001 |
| **Systolic blood pressure (per 10mmHg)** | 1.17 | (1.16, 1.18) | <0.001 | 1.23 | (1.21, 1.24) | <0.001 | 1.19 | (1.18, 1.20) | <0.001 | 1.17 | (1.17, 1.18) | <0.001 |

eGFR is measured in mL/min/1.73m^2^.

Table S2. Adjusted estimates of analysis of time to first cardiac event.

|  | **Arrhythmia** | | | **Heart failure** | | | **Ischaemic heart disease** | | | **Any cardiac event** | | |
| --- | --- | --- | --- | --- | --- | --- | --- | --- | --- | --- | --- | --- |
| **Characteristics** | *HR* | *(95% CI)* | *p* | *HR* | *(95% CI)* | *p* | *HR* | *(95% CI)* | *p* | *HR* | *(95% CI)* | *p* |
| **Creatinine** |  |  |  |  |  |  |  |  |  |  |  |  |
| **eGFRCr and sex** |  |  | 0.03 |  |  | 0.1 |  |  | 0.09 |  |  | 0.007 |
| Female |  |  |  |  |  |  |  |  |  |  |  |  |
| *<30* | 1.87 | (1.31, 2.66) |  | 3.33 | (2.07, 5.38) |  | 1.74 | (1.19, 2.54) |  | 2.28 | (1.73, 3.00) |  |
| *30-44* | 1.24 | (1.02, 1.52) |  | 1.80 | (1.31, 2.46) |  | 1.16 | (0.92, 1.46) |  | 1.20 | (1.01, 1.42) |  |
| *45-59* | 0.78 | (0.70, 0.85) |  | 0.96 | (0.80, 1.14) |  | 0.75 | (0.67, 0.83) |  | 0.80 | (0.74, 0.87) |  |
| *60-74* | 0.63 | (0.59, 0.68) |  | 0.79 | (0.69, 0.89) |  | 0.63 | (0.58, 0.68) |  | 0.66 | (0.63, 0.70) |  |
| *75-89* | 0.58 | (0.56, 0.60) |  | 0.65 | (0.60, 0.70) |  | 0.60 | (0.58, 0.63) |  | 0.61 | (0.59, 0.63) |  |
| *90-104* | 0.57 | (0.55, 0.59) |  | 0.61 | (0.57, 0.65) |  | 0.59 | (0.57, 0.61) |  | 0.60 | (0.59, 0.62) |  |
| *105+* | 0.66 | (0.61, 0.72) |  | 0.92 | (0.79, 1.08) |  | 0.58 | (0.54, 0.64) |  | 0.65 | (0.61, 0.69) |  |
| Male |  |  |  |  |  |  |  |  |  |  |  |  |
| *<30* | 2.34 | (1.80, 3.06) |  | 5.22 | (3.78, 7.21) |  | 3.21 | (2.49, 4.14) |  | 2.80 | (2.25, 3.47) |  |
| *30-44* | 1.54 | (1.28, 1.86) |  | 2.58 | (1.99, 3.35) |  | 1.35 | (1.10, 1.65) |  | 1.58 | (1.36, 1.84) |  |
| *45-59* | 1.21 | (1.10, 1.32) |  | 1.52 | (1.30, 1.77) |  | 1.24 | (1.13, 1.37) |  | 1.22 | (1.14, 1.32) |  |
| *60-74* | 1.03 | (0.97, 1.10) |  | 1.16 | (1.04, 1.30) |  | 1.07 | (1.00, 1.14) |  | 1.06 | (1.00, 1.11) |  |
| *75-89* | 0.97 | (0.94, 1.01) |  | 0.91 | (0.85, 0.98) |  | 1.02 | (0.98, 1.05) |  | 0.99 | (0.96, 1.01) |  |
| *90-104* | ref | - |  | ref | - |  | ref | - |  | ref | - |  |
| *105+* | 1.18 | (1.11, 1.27) |  | 1.55 | (1.37, 1.75) |  | 1.11 | (1.04, 1.18) |  | 1.18 | (1.12, 1.24) |  |
| **Age (per year)** | 1.09 | (1.09, 1.09) | <0.001 | 1.10 | (1.09, 1.10) | <0.001 | 1.06 | (1.06, 1.06) | <0.001 | 1.07 | (1.07, 1.08) | <0.001 |
| **Smoking Group** |  |  | <0.001 |  |  | <0.001 |  |  | <0.001 |  |  | <0.001 |
| Never | ref | - |  | ref | - |  | ref | - |  | ref | - |  |
| Previous | 1.12 | (1.09, 1.15) |  | 1.18 | (1.12, 1.24) |  | 1.14 | (1.11, 1.17) |  | 1.13 | (1.10, 1.15) |  |
| Current | 1.28 | (1.23,1.33) |  | 2.11 | (1.98, 2.26) |  | 1.64 | (1.58, 1.70) |  | 1.48 | (1.44, 1.52) |  |
| **Comorbidities** |  |  |  |  |  |  |  |  |  |  |  |  |
| Diabetes | 1.31 | (1.25, 1.37) | <0.001 | 2.03 | (1.89, 2.19) | <0.001 | 1.64 | (1.57, 1.72) | <0.001 | 1.44 | (1.39, 1.49) | <0.001 |
| Cancer | 1.15 | (1.11, 1.20) | <0.001 | 1.29 | (1.20, 1.39) | <0.001 | 1.09 | (1.04, 1.13) | <0.001 | 1.15 | (1.11, 1.18) | <0.001 |
| Hypertension | 1.49 | (1.45, 1.53) | <0.001 | 1.65 | (1.57, 1.74) | <0.001 | 1.46 | (1.42, 1.50) | <0.001 | 1.46 | (1.43, 1.49) | <0.001 |
| Hyperlipidaemia | 1.05 | (1.02, 1.08) | 0.001 | 1.15 | (1.09, 1.22) | <0.001 | 1.41 | (1.37, 1.46) | <0.001 | 1.21 | (1.18, 1.24) | <0.001 |
| **Total:HDL cholesterol** | 1.00 | (0.99, 1.01) | 0.9 | 1.08 | (1.05, 1.10) | <0.001 | 1.27 | (1.25, 1.28) | <0.001 | 1.12 | (1.11, 1.13) | <0.001 |
| **Systolic blood pressure**  **(per 10mmHg)** | 1.02 | (1.01, 1.03) | <0.001 | 1.07 | (1.06, 1.08) | <0.001 | 1.06 | (1.05, 1.07) | <0.001 | 1.04 | (1.03, 1.04) | <0.001 |

| **Cystatin C** |  |  |  |  |  |  |  |  |  |  |  |  |
| --- | --- | --- | --- | --- | --- | --- | --- | --- | --- | --- | --- | --- |
| **eGFRCys and sex** |  |  | <0.001 |  |  | <0.001 |  |  | <0.001 |  |  | <0.001 |
| Female |  |  |  |  |  |  |  |  |  |  |  |  |
| *<30* | 2.06 | (1.57, 2.70) |  | 3.88 | (2.64, 5.69) |  | 1.75 | (1.29, 2.36) |  | 2.25 | (1.81, 2.79) |  |
| *30-44* | 1.38 | (1.20, 1.58) |  | 3.01 | (2.48, 3.66) |  | 1.11 | (0.95, 1.30) |  | 1.38 | (1.23, 1.54) |  |
| *45-59* | 1.07 | (1.00, 1.15) |  | 1.89 | (1.68, 2.12) |  | 0.96 | (0.89, 1.03) |  | 1.08 | (1.03, 1.14) |  |
| *60-74* | 0.80 | (0.75, 0.84) |  | 1.16 | (1.04, 1.29) |  | 0.78 | (0.73, 0.83) |  | 0.82 | (0.78, 0.85) |  |
| *75-89* | 0.64 | (0.61, 0.67) |  | 0.76 | (0.69, 0.83) |  | 0.64 | (0.61, 0.67) |  | 0.67 | (0.65, 0.70) |  |
| *90-104* | 0.54 | (0.51, 0.57) |  | 0.52 | (0.47, 0.57) |  | 0.54 | (0.52, 0.57) |  | 0.56 | (0.54, 0.58) |  |
| *105+* | 0.45 | (0.41, 0.49) |  | 0.39 | (0.32, 0.48) |  | 0.43 | (0.39, 0.48) |  | 0.46 | (0.43, 0.49) |  |
| Male |  |  |  |  |  |  |  |  |  |  |  |  |
| *<30* | 2.96 | (2.37, 3.69) |  | 7.16 | (5.43, 9.44) |  | 2.96 | (2.37, 3.69) |  | 3.19 | (2.67, 3.82) |  |
| *30-44* | 1.96 | (1.73, 2.23) |  | 3.77 | (3.14, 4.52) |  | 1.57 | (1.37, 1.80) |  | 1.87 | (1.68, 2.07) |  |
| *45-59* | 1.53 | (1.43, 1.64) |  | 2.16 | (1.91, 2.43) |  | 1.34 | (1.25, 1.44) |  | 1.48 | (1.40, 1.56) |  |
| *60-74* | 1.35 | (1.28, 1.42) |  | 1.83 | (1.65, 2.01) |  | 1.25 | (1.18, 1.32) |  | 1.32 | (1.26, 1.37) |  |
| *75-89* | 1.12 | (1.08, 1.17) |  | 1.24 | (1.15, 1.34) |  | 1.12 | (1.08, 1.16) |  | 1.12 | (1.09, 1.15) |  |
| *90-104* | ref |  |  | ref |  |  | ref |  |  | ref |  |  |
| *105+* | 0.92 | (0.87, 0.98) |  | 0.82 | (0.72, 0.93) |  | 0.94 | (0.88, 0.99) |  | 0.94 | (0.90, 0.98) |  |
| **Age (per year)** | 1.08 | (1.08, 1.08) | <0.001 | 1.07 | (1.07, 1.08) | <0.001 | 1.05 | (1.05, 1.05) | <0.001 | 1.06 | (1.06, 1.06) | <0.001 |
| **Smoking Group** |  |  | <0.001 |  |  | <0.001 |  |  | <0.001 |  |  | <0.001 |
| Never | ref |  |  | ref |  |  | ref |  |  | ref |  |  |
| Previous | 1.12 | (1.09, 1.15) |  | 1.18 | (1.12, 1.24) |  | 1.15 | (1.12, 1.18) |  | 1.13 | (1.11, 1.15) |  |
| Current | 1.22 | (1.17, 1.26) |  | 1.90 | (1.78, 2.03) |  | 1.57 | (1.51, 1.63) |  | 1.41 | (1.37, 1.45) |  |
| **Comorbidities** |  |  |  |  |  |  |  |  |  |  |  |  |
| Diabetes | 1.28 | (1.22, 1.34) | <0.001 | 1.96 | (1.82, 2.11) | <0.001 | 1.62 | (1.55, 1.70) | <0.001 | 1.42 | (1.37, 1.47) | <0.001 |
| Cancer | 1.14 | (1.09, 1.18) | <0.001 | 1.26 | (1.17, 1.35) | <0.001 | 1.08 | (1.03, 1.12) | <0.001 | 1.13 | (1.10, 1.17) | <0.001 |
| Hypertension | 1.44 | (1.40, 1.47) | <0.001 | 1.53 | (1.45, 1.60) | <0.001 | 1.42 | (1.38, 1.46) | <0.001 | 1.41 | (1.38, 1.44) | <0.001 |
| Hyperlipidaemia | 1.03 | (1.00, 1.07) | 0.06 | 1.11 | (1.05, 1.18) | <0.001 | 1.40 | (1.36, 1.45) | <0.001 | 1.20 | (1.17, 1.23) | <0.001 |
| **Total:HDL cholesterol** | 0.97 | (0.96, 0.98) | <0.001 | 1.02 | (1.00, 1.04) | <0.001 | 1.24 | (1.23, 1.26) | <0.001 | 1.09 | (1.08, 1.10) | <0.001 |
| **Systolic blood pressure**  **(per 10mmHg)** | 1.02 | (1.02, 1.03) | <0.001 | 1.08 | (1.06, 1.09) | <0.001 | 1.06 | (1.05, 1.07) | <0.001 | 1.04 | (1.03, 1.04) | <0.001 |

| **Creatinine and cystatin C** |  |  |  |  |  |  |  |  |  |  |  |  |
| --- | --- | --- | --- | --- | --- | --- | --- | --- | --- | --- | --- | --- |
| **eGFRCr-Cys and sex** |  |  | <0.001 |  |  | <0.001 |  |  | <0.001 |  |  | <0.001 |
| Female |  |  |  |  |  |  |  |  |  |  |  |  |
| *<30* | 2.15 | (1.59, 2.90) |  | 4.19 | (2.79, 6.28) |  | 1.77 | (1.26, 2.49) |  | 2.44 | (1.91, 3.10) |  |
| *30-44* | 1.41 | (1.17, 1.70) |  | 2.51 | (1.92, 3.28) |  | 1.27 | (1.03, 1.56) |  | 1.39 | (1.20, 1.62) |  |
| *45-59* | 1.01 | (0.93, 1.10) |  | 1.68 | (1.46, 1.93) |  | 0.91 | (0.82, 1.00) |  | 1.03 | (0.96, 1.11) |  |
| *60-74* | 0.80 | (0.75, 0.85) |  | 1.15 | (1.03, 1.29) |  | 0.79 | (0.73, 0.84) |  | 0.83 | (0.79, 0.87) |  |
| *75-89* | 0.65 | (0.62, 0.67) |  | 0.78 | (0.72, 0.84) |  | 0.64 | (0.62, 0.67) |  | 0.67 | (0.65, 0.69) |  |
| *90-104* | 0.55 | (0.53, 0.57) |  | 0.53 | (0.48, 0.58) |  | 0.56 | (0.53, 0.58) |  | 0.57 | (0.56, 0.59) |  |
| *105+* | 0.54 | (0.50, 0.58) |  | 0.62 | (0.53, 0.72) |  | 0.48 | (0.44, 0.52) |  | 0.54 | (0.51, 0.57) |  |
| Male |  |  |  |  |  |  |  |  |  |  |  |  |
| *<30* | 2.58 | (2.01, 3.30) |  | 6.26 | (4.64, 8.44) |  | 2.92 | (2.29, 3.71) |  | 2.89 | (2.37, 3.53) |  |
| *30-44* | 1.93 | (1.65, 2.27) |  | 3.71 | (2.99, 4.60) |  | 1.73 | (1.46, 2.05) |  | 1.91 | (1.68, 2.17) |  |
| *45-59* | 1.48 | (1.36, 1.61) |  | 2.11 | (1.83, 2.44) |  | 1.25 | (1.14, 1.37) |  | 1.41 | (1.31, 1.51) |  |
| *60-74* | 1.25 | (1.18, 1.33) |  | 1.56 | (1.40, 1.74) |  | 1.21 | (1.14, 1.29) |  | 1.25 | (1.19, 1.31) |  |
| *75-89* | 1.11 | (1.08, 1.15) |  | 1.18 | (1.10, 1.27) |  | 1.10 | (1.06, 1.14) |  | 1.10 | (1.07, 1.13) |  |
| *90-104* | ref |  |  | ref |  |  | ref |  |  | ref |  |  |
| *105+* | 0.94 | (0.88, 1.00) |  | 0.95 | (0.83, 1.09) |  | 0.94 | (0.89, 1.00) |  | 0.97 | (0.92, 1.01) |  |
| **Age (per year)** | 1.08 | (1.08, 1.08) | <0.001 | 1.08 | (1.08, 1.09) | <0.001 | 1.05 | (1.05, 1.05) | <0.001 | 1.07 | (1.06, 1.07) | <0.001 |
| **Smoking Group** |  |  | <0.001 |  |  | <0.001 |  |  | <0.001 |  |  | <0.001 |
| Never | ref |  |  | ref |  |  | ref |  |  | ref |  |  |
| Previous | 1.12 | (1.09, 1.15) |  | 1.18 | (1.12, 1.24) |  | 1.15 | (1.12, 1.18) |  | 1.13 | (1.11, 1.15) |  |
| Current | 1.27 | (1.22, 1.32) |  | 2.07 | (1.94, 2.21) |  | 1.62 | (1.56, 1.68) |  | 1.46 | (1.42, 1.51) |  |
| **Comorbidities** |  |  |  |  |  |  |  |  |  |  |  |  |
| Diabetes | 1.31 | (1.25, 1.37) | <0.001 | 2.03 | (1.89, 2.18) | <0.001 | 1.64 | (1.57, 1.72) | <0.001 | 1.44 | (1.39, 1.49) | <0.001 |
| Cancer | 1.14 | (1.10, 1.19) | <0.001 | 1.27 | (1.18, 1.36) | <0.001 | 1.08 | (1.04, 1.13) | <0.001 | 1.14 | (1.11, 1.17) | <0.001 |
| Hypertension | 1.46 | (1.42, 1.50) | <0.001 | 1.58 | (1.50, 1.66) | <0.001 | 1.44 | (1.40, 1.48) | <0.001 | 1.43 | (1.40, 1.46) | <0.001 |
| Hyperlipidaemia | 1.04 | (1.00, 1.07) | 0.03 | 1.12 | (1.06, 1.19) | <0.001 | 1.40 | (1.36, 1.45) | <0.001 | 1.20 | (1.17, 1.23) | <0.001 |
| **Total:HDL cholesterol** | 0.99 | (0.98, 1.00) | 0.01 | 1.05 | (1.03, 1.07) | <0.001 | 1.25 | (1.24, 1.27) | <0.001 | 1.11 | (1.10, 1.12) | <0.001 |
| **Systolic blood pressure**  **(per 10mmHg)** | 1.02 | (1.02, 1.03) | <0.001 | 1.08 | (1.06, 1.09) | <0.001 | 1.06 | (1.05, 1.07) | <0.001 | 1.04 | (1.03, 1.04) | <0.001 |

eGFR is measured in mL/min/1.73m^2^

Table S3. Comparison of AIC and C-statistic for adjusted models.

| **Outcome** | **eGFR measure** | **AIC** | **C-statistic** |
| --- | --- | --- | --- |
| Any cardiac event | Creatinine | 1,200,161 | 0.71 |
|  | Cystatin C | 1,199,204 | 0.71 |
|  | Creatinine and cystatin C | 1,199,698 | 0.71 |
| Cardiac death | Creatinine | 89,329 | 0.80 |
|  | Cystatin C | 88,967 | 0.81 |
|  | Creatinine and cystatin C | 89,168 | 0.81 |

Table S4. Unadjusted estimates of analysis of time to cardiac death.

|  | **Arrythmia** | | | **Heart failure** | | | **Ischaemic heart disease** | | | **Any cardiac disease** | | |
| --- | --- | --- | --- | --- | --- | --- | --- | --- | --- | --- | --- | --- |
| **Characteristic** | *HR* | *(95% CI)* | *p* | *HR* | *(95% CI)* | *p* | *HR* | *(95% CI)* | *p* | *HR* | *(95% CI)* | *p* |
| **eGFRCr and sex** |  |  | 0.5 |  |  | 0.2 |  |  | 0.004 |  |  | 0.005 |
| Female |  |  |  |  |  |  |  |  |  |  |  |  |
| *<30* | 15.09 | (6.22, 36.62) |  | 8.22 | (2.63, 25.66) |  | 9.94 | (5.75, 17.19) |  | 11.23 | (7.13, 17.66) |  |
| *30-44* | 2.31 | (0.74, 7.21) |  | 4.92 | (2.32, 10.44) |  | 3.26 | (2.02, 5.27) |  | 3.81 | (2.59, 5.62) |  |
| *45-59* | 1.38 | (0.80, 2.36) |  | 1.52 | (0.93, 2.49) |  | 0.92 | (0.66, 1.27) |  | 1.19 | (0.93, 1.53) |  |
| *60-74* | 0.99 | (0.67, 1.46) |  | 1.36 | (0.99, 1.88) |  | 0.45 | (0.34, 0.59) |  | 0.69 | (0.57, 0.84) |  |
| *75-89* | 0.61 | (0.49, 0.77) |  | 0.51 | (0.41, 0.64) |  | 0.28 | (0.24, 0.33) |  | 0.38 | (0.34, 0.43) |  |
| *90-104* | 0.47 | (0.38, 0.59) |  | 0.42 | (0.34, 0.52) |  | 0.26 | (0.23, 0.30) |  | 0.33 | (0.30, 0.37) |  |
| *105+* | 0.14 | (0.07, 0.25) |  | 0.28 | (0.19, 0.43) |  | 0.16 | (0.12, 0.21) |  | 0.18 | (0.15, 0.23) |  |
| Male |  |  |  |  |  |  |  |  |  |  |  |  |
| *<30* | 11.89 | (4.90, 28.86) |  | 8.69 | (3.24, 23.35) |  | 10.51 | (6.50, 16.98) |  | 11.87 | (7.99, 17.64) |  |
| *30-44* | 8.98 | (4.77, 16.93) |  | 7.37 | (3.79, 14.33) |  | 7.25 | (5.10, 10.32) |  | 8.80 | (6.63, 11.68) |  |
| *45-59* | 3.48 | (2.27, 5.35) |  | 4.69 | (3.28, 6.72) |  | 3.14 | (2.51, 3.93) |  | 3.52 | (2.92, 4.24) |  |
| *60-74* | 2.51 | (1.85, 3.40) |  | 2.01 | (1.46, 2.76) |  | 1.71 | (1.43, 2.04) |  | 1.98 | (1.71, 2.29) |  |
| *75-89* | 1.44 | (1.19, 1.73) |  | 1.31 | (1.09, 1.57) |  | 1.21 | (1.10, 1.33) |  | 1.26 | (1.16, 1.37) |  |
| *90-104* | ref | - |  | ref | - |  | ref | - |  | ref | - |  |
| *105+* | 0.45 | (0.30, 0.66) |  | 0.72 | (0.53, 0.97) |  | 0.76 | (0.65, 0.89) |  | 0.73 | (0.64, 0.84) |  |
| **eGFRCr** |  |  | <0.001 |  |  | <0.001 |  |  | <0.001 |  |  | <0.001 |
| *<30* | 18.85 | (10.05, 35.33) |  | 12.52 | (5.93, 26.45) |  | 17.49 | (12.17, 25.12) |  | 18.51 | (13.72, 24.97) |  |
| *30-44* | 7.63 | (4.39, 13.27) |  | 8.94 | (5.42, 14.74) |  | 8.68 | (6.52, 11.55) |  | 9.72 | (7.72, 12.23) |  |
| *45-59* | 3.12 | (2.23, 4.38) |  | 4.09 | (3.05, 5.47) |  | 3.05 | (2.53, 3.68) |  | 3.36 | (2.89, 3.91) |  |
| *60-74* | 2.28 | (1.79, 2.91) |  | 2.40 | (1.91, 3.03) |  | 1.65 | (1.42, 1.91) |  | 1.94 | (1.73, 2.19) |  |
| *75-89* | 1.40 | (1.20, 1.62) |  | 1.28 | (1.11, 1.49) |  | 1.19 | (1.10, 1.30) |  | 1.24 | (1.16, 1.33) |  |
| *90-104* | ref | - |  | ref | - |  | ref | - |  | ref | - |  |
| *105+* | 0.39 | (0.28, 0.54) |  | 0.70 | (0.54, 0.89) |  | 0.72 | (0.62, 0.82) |  | 0.67 | (0.60, 0.76) |  |
| **eGFRCys and sex** |  |  | 0.003 |  |  | 0.003 |  |  | <0.001 |  |  | <0.001 |
| Female |  |  |  |  |  |  |  |  |  |  |  |  |
| *<30* | 19.13 | (9.32, 39.25) |  | 17.62 | (8.19, 37.91) |  | 12.68 | (8.19, 19.65) |  | 16.59 | (11.79, 23.33) |  |
| *30-44* | 3.22 | (1.50, 6.92) |  | 11.73 | (7.52, 18.30) |  | 4.34 | (3.11, 6.04) |  | 5.40 | (4.16, 7.02) |  |
| *45-59* | 3.13 | (2.22, 4.41) |  | 4.19 | (3.05, 5.77) |  | 1.56 | (1.25, 1.94) |  | 2.23 | (1.89, 2.64) |  |
| *60-74* | 1.78 | (1.30, 2.42) |  | 1.88 | (1.37, 2.56) |  | 0.77 | (0.63, 0.94) |  | 1.06 | (0.91, 1.25) |  |
| *75-89* | 0.82 | (0.63, 1.06) |  | 0.84 | (0.64, 1.09) |  | 0.42 | (0.36, 0.49) |  | 0.54 | (0.48, 0.61) |  |
| *90-104* | 0.32 | (0.23, 0.45) |  | 0.28 | (0.19, 0.40) |  | 0.19 | (0.16, 0.23) |  | 0.23 | (0.20, 0.27) |  |
| *105+* | 0.06 | (0.02, 0.15) |  | 0.13 | (0.07, 0.26) |  | 0.08 | (0.05, 0.12) |  | 0.10 | (0.07, 0.13) |  |
| Male |  |  |  |  |  |  |  |  |  |  |  |  |
| *<30* | 21.42 | (11.20, 40.97) |  | 22.58 | (11.79, 43.24) |  | 17.30 | (12.02, 24.90) |  | 20.32 | (15.09, 27.36) |  |
| *30-44* | 11.76 | (7.37, 18.79) |  | 17.27 | (11.43, 26.10) |  | 9.39 | (7.26, 12.15) |  | 11.39 | (9.25, 14.02) |  |
| *45-59* | 6.73 | (4.97, 9.12) |  | 7.38 | (5.45, 9.98) |  | 4.89 | (4.14, 5.77) |  | 5.47 | (4.76, 6.29) |  |
| *60-74* | 3.84 | (2.93, 5.02) |  | 4.82 | (3.71, 6.25) |  | 3.00 | (2.61, 3.46) |  | 3.24 | (2.87, 3.66) |  |
| *75-89* | 1.55 | (1.23, 1.96) |  | 1.82 | (1.44, 2.29) |  | 1.61 | (1.43, 1.80) |  | 1.59 | (1.44, 1.76) |  |
| *90-104* | ref | - |  | ref | - |  | ref | - |  | ref | - |  |
| *105+* | 0.53 | (0.36, 0.79) |  | 0.44 | (0.29, 0.68) |  | 0.54 | (0.44, 0.65) |  | 0.53 | (0.45, 0.63) |  |
| **eGFRCys** |  |  | <0.001 |  |  | <0.001 |  |  | <0.001 |  |  | <0.001 |
| *<30* | 34.10 | (20.94, 55.55) |  | 35.62 | (21.54, 58.90) |  | 29.14 | (21.93, 38.72) |  | 34.25 | (27.27, 43.00) |  |
| *30-44* | 11.86 | (7.93, 17.74) |  | 25.04 | (18.27, 34.33) |  | 12.76 | (10.36, 15.72) |  | 14.91 | (12.61, 17.62) |  |
| *45-59* | 7.70 | (6.05, 9.79) |  | 9.65 | (7.62, 12.23) |  | 5.60 | (4.87, 6.45) |  | 6.53 | (5.83, 7.32) |  |
| *60-74* | 4.48 | (3.60, 5.56) |  | 5.55 | (4.47, 6.89) |  | 3.35 | (2.96, 3.79) |  | 3.70 | (3.34, 4.10) |  |
| *75-89* | 1.95 | (1.61, 2.35) |  | 2.28 | (1.88, 2.77) |  | 1.88 | (1.70, 2.08) |  | 1.90 | (1.75, 2.07) |  |
| *90-104* | ref | - |  | ref | - |  | ref | - |  | ref | - |  |
| *105+* | 0.47 | (0.33, 0.67) |  | 0.49 | (0.34, 0.70) |  | 0.57 | (0.48, 0.68) |  | 0.55 | (0.47, 0.64) |  |
| **eGFRCr-Cys and sex** |  |  | 0.03 |  |  | 0.08 |  |  | <0.001 |  |  | <0.001 |
| Female |  |  |  |  |  |  |  |  |  |  |  |  |
| *<30* | 18.49 | (8.17, 41.84) |  | 20.00 | (8.83, 45.29) |  | 11.67 | (6.99, 19.48) |  | 16.04 | (10.85, 23.72) |  |
| *30-44* | 3.47 | (1.28, 9.37) |  | 4.63 | (1.90, 11.31) |  | 5.20 | (3.46, 7.82) |  | 5.16 | (3.60, 7.39) |  |
| *45-59* | 3.46 | (2.29, 5.21) |  | 6.17 | (4.40, 8.63) |  | 1.72 | (1.30, 2.26) |  | 2.75 | (2.26, 3.34) |  |
| *60-74* | 1.60 | (1.12, 2.28) |  | 1.93 | (1.37, 2.72) |  | 0.78 | (0.61, 0.99) |  | 1.10 | (0.92, 1.32) |  |
| *75-89* | 0.94 | (0.75, 1.17) |  | 0.99 | (0.78, 1.24) |  | 0.44 | (0.39, 0.51) |  | 0.60 | (0.54, 0.67) |  |
| *90-104* | 0.36 | (0.27, 0.48) |  | 0.32 | (0.24, 0.44) |  | 0.22 | (0.18, 0.26) |  | 0.27 | (0.23, 0.31) |  |
| *105+* | 0.14 | (0.08, 0.26) |  | 0.29 | (0.18, 0.46) |  | 0.14 | (0.10, 0.19) |  | 0.17 | (0.14, 0.22) |  |
| Male |  |  |  |  |  |  |  |  |  |  |  |  |
| *<30* | 15.08 | (6.66, 34.12) |  | 18.95 | (8.86, 40.50) |  | 15.13 | (9.97, 22.94) |  | 16.27 | (11.42, 23.17) |  |
| *30-44* | 11.91 | (6.76, 21.01) |  | 14.78 | (8.67, 25.18) |  | 8.90 | (6.44, 12.31) |  | 11.86 | (9.23, 15.25) |  |
| *45-59* | 6.43 | (4.40, 9.38) |  | 8.97 | (6.37, 12.64) |  | 5.24 | (4.27, 6.43) |  | 5.95 | (5.02, 7.05) |  |
| *60-74* | 5.02 | (3.84, 6.57) |  | 4.78 | (3.61, 6.33) |  | 3.32 | (2.85, 3.87) |  | 3.81 | (3.35, 4.33) |  |
| *75-89* | 1.77 | (1.45, 2.17) |  | 2.23 | (1.82, 2.73) |  | 1.71 | (1.54, 1.89) |  | 1.75 | (1.60, 1.91) |  |
| *90-104* | ref | - |  | ref | - |  | ref | - |  | ref | - |  |
| *105+* | 0.51 | (0.34, 0.76) |  | 0.66 | (0.45, 0.97) |  | 0.65 | (0.54, 0.79) |  | 0.64 | (0.54, 0.75) |  |
| **eGFRCr-Cys** |  |  | <0.001 |  |  | <0.001 |  |  | <0.001 |  |  | <0.001 |
| *<30* | 25.73 | (14.38, 46.03) |  | 31.04 | (17.70, 54.44) |  | 23.88 | (17.24, 33.08) |  | 27.31 | (20.96, 35.57) |  |
| *30-44* | 11.73 | (7.16, 19.23) |  | 15.27 | (9.63, 24.20) |  | 12.36 | (9.56, 15.98) |  | 14.16 | (11.50, 17.43) |  |
| *45-59* | 7.18 | (5.39, 9.56) |  | 11.63 | (9.04, 14.97) |  | 5.48 | (4.62, 6.48) |  | 6.76 | (5.92, 7.73) |  |
| *60-74* | 4.66 | (3.73, 5.83) |  | 4.97 | (3.95, 6.25) |  | 3.22 | (2.81, 3.67) |  | 3.74 | (3.35, 4.17) |  |
| *75-89* | 2.04 | (1.73, 2.41) |  | 2.48 | (2.10, 2.94) |  | 1.80 | (1.64, 1.96) |  | 1.90 | (1.76, 2.05) |  |
| *90-104* | ref | - |  | ref | - |  | ref | - |  | ref | - |  |
| *105+* | 0.45 | (0.32, 0.63) |  | 0.70 | (0.52, 0.95) |  | 0.62 | (0.52, 0.72) |  | 0.61 | (0.53, 0.70) |  |
| **Female** | 0.44 | (0.38, 0.50) | <0.001 | 0.43 | (0.38, 0.49) | <0.001 | 0.25 | (0.23, 0.28) | <0.001 | 0.32 | (0.30, 0.35) | <0.001 |
| **Age (per year)** | 1.15 | (1.14, 1.16) | <0.001 | 1.13 | (1.12, 1.14) | <0.001 | 1.10 | (1.10, 1.11) | <0.001 | 1.11 | (1.11, 1.12) | <0.001 |
| **Smoker** |  |  | <0.001 |  |  | <0.001 |  |  | <0.001 |  |  |  |
| Never | ref | - |  | ref | - |  | ref | - |  | ref | - |  |
| Previous | 1.76 | (1.52, 2.04) |  | 1.60 | (1.38, 1.85) |  | 1.82 | (1.67, 1.98) |  | 1.79 | (1.67, 1.92) | <0.001 |
| Current | 2.40 | (1.99, 2.90) |  | 3.05 | (2.58, 3.61) |  | 3.48 | (3.15, 3.83) |  | 3.16 | (2.91, 3.43) | <0.001 |
| **Comorbidities** |  |  |  |  |  |  |  |  |  |  |  |  |
| Diabetes | 4.21 | (3.51, 5.05) | <0.001 | 5.02 | (4.26, 5.93) | <0.001 | 4.75 | (4.31, 5.24) | <0.001 | 4.69 | (4.33, 5.09) | <0.001 |
| Cancer | 1.71 | (1.39, 2.10) | <0.001 | 1.85 | (1.53, 2.24) | <0.001 | 1.35 | (1.19, 1.53) | <0.001 | 1.54 | (1.40, 1.71) | <0.001 |
| Hypertension | 3.02 | (2.65, 3.45) | <0.001 | 3.13 | (2.75, 3.56) | <0.001 | 2.78 | (2.58, 2.99) | <0.001 | 2.83 | (2.67, 3.01) | <0.001 |
| Hyperlipidaemia | 3.06 | (2.65, 3.53) | <0.001 | 1.15 | (2.75, 3.62) | <0.001 | 2.98 | (2.75, 3.22) | <0.001 | 2.99 | (2.79, 3.19) | <0.001 |
| **Total:HDL cholesterol** | 1.00 | (0.94, 1.07) | 0.9 | 1.10 | (1.04, 1.16) | 0.001 | 1.29 | (1.25, 1.33) | <0.001 | 1.20 | (1.16, 1.22) | <0.001 |
| **Systolic blood pressure (per 10mmHg)** | 1.26 | (1.22, 1.29) | <0.001 | 1.22 | (1.19, 1.26) | <0.001 | 1.27 | (1.25, 1.30) | <0.001 | 1.26 | (1.24, 1.27) | <0.001 |

eGFR is measured in mL/min/1.73m^2^.

Table S5. Adjusted estimates of analysis of time to cardiac death

|  | **Arrhythmia** | | | **Heart failure** | | | **Ischaemic heart disease** | | | **Any cardiac death** | | |
| --- | --- | --- | --- | --- | --- | --- | --- | --- | --- | --- | --- | --- |
| **Characteristics** | *HR* | *(95% CI)* | *p* | *HR* | *(95% CI)* | *p* | *HR* | *(95% CI)* | *p* | *HR* | *(95% CI)* | *p* |
| **Creatinine** |  |  |  |  |  |  |  |  |  |  |  |  |
| **eGFRCr and sex** |  |  | 0.6 |  |  | 0.3 |  |  | 0.07 |  |  | 0.06 |
| Female |  |  |  |  |  |  |  |  |  |  |  |  |
| *<30* | 6.66 | (2.47, 18.01) |  | 4.35 | (1.39, 13.64) |  | 4.89 | (2.61, 9.16) |  | 5.99 | (3.59, 9.99) |  |
| *30-44* | 0.73 | (0.18, 2.96) |  | 2.02 | (0.89, 4.56) |  | 1.77 | (1.06, 2.96) |  | 1.93 | (1.27, 2.92) |  |
| *45-59* | 0.64 | (0.35, 1.18) |  | 0.76 | (0.44, 1.31) |  | 0.66 | (0.47, 0.93) |  | 0.74 | (0.57, 0.97) |  |
| *60-74* | 0.69 | (0.46, 1.03) |  | 0.88 | (0.62, 1.26) |  | 0.39 | (0.29, 0.52) |  | 0.55 | (0.45, 0.68) |  |
| *75-89* | 0.55 | (0.43, 0.70) |  | 0.49 | (0.38, 0.62) |  | 0.31 | (0.27, 0.36) |  | 0.40 | (0.35, 0.45) |  |
| *90-104* | 0.50 | (0.40, 0.63) |  | 0.47 | (0.37, 0.59) |  | 0.34 | (0.29, 0.39) |  | 0.41 | (0.36, 0.46) |  |
| *105+* | 0.46 | (0.22, 0.94) |  | 1.05 | (0.66, 1.67) |  | 0.55 | (0.41, 0.75) |  | 0.65 | (0.51, 0.84) |  |
| Male |  |  |  |  |  |  |  |  |  |  |  |  |
| *<30* | 4.96 | (2.03, 12.10) |  | 2.63 | (0.84, 8.26) |  | 5.04 | (3.06, 8.29) |  | 5.03 | (3.29, 7.68) |  |
| *30-44* | 3.18 | (1.62, 6.24) |  | 2.76 | (1.41, 5.40) |  | 2.74 | (1.86, 4.03) |  | 3.43 | (2.54, 4.64) |  |
| *45-59* | 1.56 | (1.00, 2.44) |  | 2.11 | (1.46, 3.06) |  | 1.56 | (1.23, 1.98) |  | 1.70 | (1.40, 2.06) |  |
| *60-74* | 1.33 | (0.96, 1.84) |  | 1.20 | (0.87, 1.67) |  | 1.06 | (0.88, 1.28) |  | 1.20 | (1.03, 1.41) |  |
| *75-89* | 1.05 | (0.86, 1.28) |  | 0.95 | (0.79, 1.16) |  | 0.97 | (0.87, 1.07) |  | 0.98 | (0.90, 1.07) |  |
| *90-104* | ref | - |  | ref | - |  | ref | - |  | ref | - |  |
| *105+* | 1.40 | (0.91, 2.15) |  | 1.98 | (1.41, 2.79) |  | 1.76 | (1.47, 2.09) |  | 1.80 | (1.55, 2.11) |  |
| **Age (per year)** | 1.13 | (1.11, 1.14) | <0.001 | 1.12 | (1.11, 1.14) | <0.001 | 1.09 | (1.08, 1.10) | <0.001 | 1.10 | (1.09, 1.11) | <0.001 |
| **Smoking Group** |  |  | <0.001 |  |  | <0.001 |  |  | <0.001 |  |  | <0.001 |
| Never | ref | - |  | ref | - |  | ref | - |  | ref | - |  |
| Previous | 1.34 | (1.15, 1.57) |  | 1.15 | (0.99, 1.35) | 0.07 | 1.27 | (1.16, 1.39) |  | 1.29 | (1.20, 1.39) |  |
| Current | 2.71 | (2.22, 3.31) |  | 3.04 | (2.54, 3.64) | <0.001 | 3.12 | (2.81, 3.46) |  | 3.05 | (2.79, 3.33) |  |
| **Comorbidities** |  |  |  |  |  |  |  |  |  |  |  |  |
| Diabetes | 2.20 | (1.78, 2.73) | <0.001 | 2.66 | (2.19, 3.24) | <0.001 | 2.57 | (2.29, 2.89) | <0.001 | 2.55 | (2.31, 2.81) | <0.001 |
| Cancer | 1.25 | (1.01, 1.55) | 0.04 | 1.33 | (1.08, 1.63) | 0.01 | 1.09 | (0.95, 1.24) | 0.2 | 1.19 | (1.08, 1.33) | <0.001 |
| Hypertension | 1.63 | (1.40, 1.91) | <0.001 | 1.68 | (1.44, 1.95) | <0.001 | 1.49 | (1.37, 1.62) | <0.001 | 1.52 | (1.42, 1.64) | <0.001 |
| Hyperlipidaemia | 1.04 | (0.87, 1.24) | 0.7 | 1.12 | (0.94, 1.33) | 0.2 | 1.20 | (1.08, 1.33) | <0.001 | 1.15 | (1.05, 1.25) | 0.002 |
| **Total:HDL cholesterol** | 0.96 | (0.90, 1.03) | 0.2 | 1.06 | (1.00, 1.13) | 0.05 | 1.21 | (1.17, 1.25) | <0.001 | 1.13 | (1.10, 1.17) | <0.001 |
| **Systolic blood pressure**  **(per 10mmHg)** | 1.07 | (1.04, 1.11) | <0.001 | 1.05 | (1.01, 1.08) | 0.02 | 1.12 | (1.10, 1.14) | <0.001 | 1.10 | (1.08, 1.12) | <0.001 |

| **Cystatin C** |  |  |  |  |  |  |  |  |  |  |  |  |
| --- | --- | --- | --- | --- | --- | --- | --- | --- | --- | --- | --- | --- |
| **eGFRCys and sex** |  |  | 0.07 |  |  | 0.05 |  |  | <0.001 |  |  | <0.001 |
| Female |  |  |  |  |  |  |  |  |  |  |  |  |
| *<30* | 7.66 | (3.53, 16.61) |  | 7.95 | (3.66, 17.27) |  | 5.41 | (3.32, 8.84) |  | 7.66 | (3.53, 16.61) |  |
| *30-44* | 1.15 | (0.50, 2.63) |  | 3.93 | (2.38, 6.50) |  | 1.91 | (1.35, 2.72) |  | 1.15 | (0.50, 2.63) |  |
| *45-59* | 1.44 | (0.99, 2.07) |  | 1.99 | (1.41, 2.81) |  | 0.85 | (0.67, 1.07) |  | 1.44 | (0.99, 2.07) |  |
| *60-74* | 0.98 | (0.70, 1.36) |  | 1.11 | (0.80, 1.55) |  | 0.51 | (0.41, 0.63) |  | 0.98 | (0.70, 1.36) |  |
| *75-89* | 0.57 | (0.43, 0.75) |  | 0.62 | (0.46, 0.82) |  | 0.37 | (0.31, 0.43) |  | 0.57 | (0.43, 0.75) |  |
| *90-104* | 0.35 | (0.24, 0.50) |  | 0.30 | (0.20, 0.44) |  | 0.24 | (0.20, 0.30) |  | 0.35 | (0.24, 0.50) |  |
| *105+* | 0.13 | (0.04, 0.41) |  | 0.32 | (0.15, 0.66) |  | 0.25 | (0.16, 0.38) |  | 0.13 | (0.04, 0.41) |  |
| Male |  |  |  |  |  |  |  |  |  |  |  |  |
| *<30* | 8.61 | (4.32, 17.19) |  | 8.96 | (4.49, 17.91) |  | 7.29 | (4.92, 10.82) |  | 8.61 | (4.32, 17.19) |  |
| *30-44* | 3.97 | (2.43, 6.51) |  | 6.14 | (3.99, 9.45) |  | 2.96 | (2.23, 3.94) |  | 3.97 | (2.43, 6.51) |  |
| *45-59* | 2.55 | (1.83, 3.55) |  | 2.84 | (2.04, 3.95) |  | 1.97 | (1.65, 2.37) |  | 2.55 | (1.83, 3.55) |  |
| *60-74* | 1.95 | (1.46, 2.59) |  | 2.45 | (1.85, 3.24) |  | 1.53 | (1.32, 1.79) |  | 1.95 | (1.46, 2.59) |  |
| *75-89* | 1.10 | (0.86, 1.41) |  | 1.33 | (1.04, 1.70) |  | 1.15 | (1.02, 1.30) |  | 1.10 | (0.86, 1.41) |  |
| *90-104* | ref | - |  | ref | - |  | ref | - |  | ref | - |  |
| *105+* | 0.84 | (0.55, 1.28) |  | 0.64 | (0.40, 1.01) |  | 0.81 | (0.66, 0.99) |  | 0.84 | (0.55, 1.28) |  |
| **Age (per year)** | 1.10 | (1.09, 1.12) | <0.001 | 1.08 | (1.07, 1.09) | <0.001 | 1.07 | (1.06, 1.07) | <0.001 | 1.10 | (1.09, 1.12) | <0.001 |
| **Smoking Group** |  |  | <0.001 |  |  | <0.001 |  |  | <0.001 |  |  | <0.001 |
| Never | ref | - |  | ref | - |  | ref | - |  | ref | - |  |
| Previous | 1.34 | (1.15, 1.57) |  | 1.15 | (0.99, 1.35) | 0.07 | 1.28 | (1.17, 1.40) |  | 1.34 | (1.15, 1.57) |  |
| Current | 2.35 | (1.92, 2.87) |  | 2.59 | (2.16, 3.11) | <0.001 | 2.88 | (2.60, 3.20) |  | 2.35 | (1.92, 2.87) |  |
| **Comorbidities** |  |  |  |  |  |  |  |  |  |  |  |  |
| Diabetes | 2.04 | (1.65, 2.53) | <0.001 | 2.47 | (2.03, 3.01) | <0.001 | 2.53 | (2.25, 2.84) | <0.001 | 2.04 | (1.65, 2.53) | <0.001 |
| Cancer | 1.20 | (0.97, 1.49) | 0.09 | 1.26 | (1.03, 1.55) | 0.03 | 1.06 | (0.93, 1.21) | 0.4 | 1.20 | (0.97, 1.49) | 0.09 |
| Hypertension | 1.48 | (1.27, 1.73) | <0.001 | 1.48 | (1.27, 1.72) | <0.001 | 1.40 | (1.29, 1.53) | <0.001 | 1.48 | (1.27, 1.73) | <0.001 |
| Hyperlipidaemia | 1.00 | (0.84, 1.20) | 0.9 | 1.06 | (0.89, 1.27) | 0.5 | 1.17 | (1.06, 1.30) | 0.002 | 1.00 | (0.84, 1.20) | 0.9 |
| **Total:HDL cholesterol** | 0.90 | (0.84, 0.97) | 0.003 | 0.99 | (0.93, 1.05) | 0.6 | 1.16 | (1.12, 1.20) | <0.001 | 0.90 | (0.84, 0.97) | 0.003 |
| **Systolic blood pressure**  **(per 10mmHg)** | 1.08 | (1.04, 1.12) | <0.001 | 1.06 | (1.02, 1.09) | 0.003 | 1.13 | (1.11, 1.15) | <0.001 | 1.11 | (1.09, 1.12) | <0.001 |

| **Creatinine and cystatin C** |  |  |  |  |  |  |  |  |  |  |  |  |
| --- | --- | --- | --- | --- | --- | --- | --- | --- | --- | --- | --- | --- |
| **eGFRCr-Cys and sex** |  |  | 0.1 |  |  | 0.03 |  |  | 0.003 |  |  | <0.001 |
| Female |  |  |  |  |  |  |  |  |  |  |  |  |
| *<30* | 6.74 | (2.74, 16.57) |  | 8.76 | (3.83, 20.02) |  | 4.42 | (2.42, 8.06) |  | 6.74 | (2.74, 16.57) |  |
| *30-44* | 1.37 | (0.51, 3.73) |  | 1.47 | (0.54, 4.00) |  | 2.44 | (1.60, 3.72) |  | 1.37 | (0.51, 3.73) |  |
| *45-59* | 1.20 | (0.75, 1.91) |  | 2.47 | (1.70, 3.58) |  | 0.85 | (0.63, 1.14) |  | 1.20 | (0.75, 1.91) |  |
| *60-74* | 0.90 | (0.63, 1.30) |  | 1.02 | (0.70, 1.47) |  | 0.51 | (0.40, 0.66) |  | 0.90 | (0.63, 1.30) |  |
| *75-89* | 0.64 | (0.50, 0.81) |  | 0.74 | (0.58, 0.95) |  | 0.39 | (0.34, 0.45) |  | 0.64 | (0.50, 0.81) |  |
| *90-104* | 0.36 | (0.26, 0.49) |  | 0.34 | (0.24, 0.48) |  | 0.28 | (0.23, 0.34) |  | 0.36 | (0.26, 0.49) |  |
| *105+* | 0.34 | (0.18, 0.65) |  | 0.66 | (0.40, 1.08) |  | 0.35 | (0.26, 0.49) |  | 0.34 | (0.18, 0.65) |  |
| Male |  |  |  |  |  |  |  |  |  |  |  |  |
| *<30* | 4.90 | (2.00, 12.04) |  | 6.33 | (2.78, 14.45) |  | 6.20 | (4.00, 9.63) |  | 4.90 | (2.00, 12.04) |  |
| *30-44* | 4.02 | (2.26, 7.17) |  | 4.98 | (2.89, 8.59) |  | 3.00 | (2.12, 4.25) |  | 4.02 | (2.26, 7.17) |  |
| *45-59* | 2.16 | (1.44, 3.24) |  | 3.13 | (2.17, 4.51) |  | 1.98 | (1.59, 2.47) |  | 2.16 | (1.44, 3.24) |  |
| *60-74* | 2.14 | (1.60, 2.85) |  | 2.14 | (1.58, 2.89) |  | 1.55 | (1.32, 1.83) |  | 2.14 | (1.60, 2.85) |  |
| *75-89* | 1.11 | (0.90, 1.38) |  | 1.45 | (1.17, 1.79) |  | 1.14 | (1.02, 1.27) |  | 1.11 | (0.90, 1.38) |  |
| *90-104* | ref | - |  | ref | - |  | ref | - |  | ref | - |  |
| *105+* | 0.91 | (0.58, 1.43) |  | 1.16 | (0.77, 1.74) |  | 1.07 | (0.88, 1.30) |  | 0.91 | (0.58, 1.43) |  |
| **Age (per year)** | 1.11 | (1.09, 1.13) | <0.001 | 1.09 | (1.08, 1.11) | <0.001 | 1.07 | (1.07, 1.08) | <0.001 | 1.11 | (1.09, 1.13) | <0.001 |
| **Smoking Group** |  |  | <0.001 |  |  | <0.001 |  |  | <0.001 |  |  | <0.001 |
| Never | ref | - |  | ref | - |  | ref | - |  | ref | - |  |
| Previous | 1.34 | (1.15, 1.57) |  | 1.16 | (0.99, 1.35) | 0.06 | 1.28 | (1.17, 1.40) |  | 1.34 | (1.15, 1.57) |  |
| Current | 2.59 | (2.12, 3.16) |  | 2.93 | (2.45, 3.51) | <0.001 | 3.10 | (2.79, 3.43) |  | 2.59 | (2.12, 3.16) |  |
| **Comorbidities** |  |  |  |  |  |  |  |  |  |  |  |  |
| Diabetes | 2.15 | (1.74, 2.66) | <0.001 | 2.63 | (2.17, 3.21) | <0.001 | 2.59 | (2.30, 2.91) | <0.001 | 2.15 | (1.74, 2.66) | <0.001 |
| Cancer | 1.22 | (0.98, 1.51) | 0.07 | 1.28 | (1.04, 1.58) | 0.02 | 1.07 | (0.94, 1.22) | 0.3 | 1.22 | (0.98, 1.51) | 0.07 |
| Hypertension | 1.54 | (1.32, 1.80) | <0.001 | 1.56 | (1.34, 1.82) | <0.001 | 1.44 | (1.32, 1.57) | <0.001 | 1.54 | (1.32, 1.80) | <0.001 |
| Hyperlipidaemia | 1.01 | (0.85, 1.21) | 0.01 | 1.08 | (0.91, 1.29) | 0.4 | 1.18 | (1.07, 1.31) | 0.001 | 1.01 | (0.85, 1.21) | 0.9 |
| **Total:HDL cholesterol** | 0.93 | (0.87, 0.99) | 0.03 | 1.02 | (0.96, 1.09) | 0.5 | 1.19 | (1.15, 1.23) | <0.001 | 0.93 | (0.87, 0.99) | 0.03 |
| **Systolic blood pressure**  **(per 10mmHg)** | 1.08 | (1.04, 1.12) | <0.001 | 1.05 | (1.02, 1.09) | 0.004 | 1.13 | (1.10, 1.15) | <0.001 | 1.10 | (1.09, 1.12) | <0.001 |

eGFR is measured in mL/min/1.73m^2^
