# Supplementary material for: Sex differences in cardiac risk and kidney function: serum creatinine versus cystatin C
Source: BMC Med. 2025 Dec 30;24:62. doi: 10.1186/s12916-025-04588-9 (PMC12860083; doi:10.1186/s12916-025-04588-9)
Supplement: Supplementary file 2 — Additional file 2. Figure S1: Calibration plots for adjusted models for analysis of time to: (A) first cardiac event and (B) first cardiac death [file 12916_2025_4588_MOESM2_ESM.docx]

Figure S1. Calibration plots for adjusted models for analysis of time to: (A) first cardiac event, (B) first cardiac death and (C) first cardiac event or death.

**(A) First cardiac event**

**
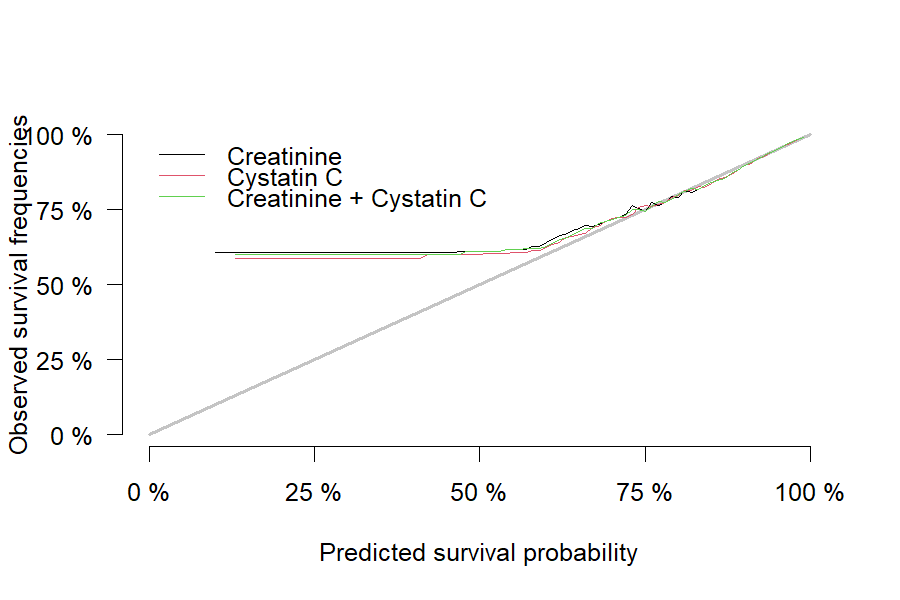
**

**(B) First cardiac death**

**
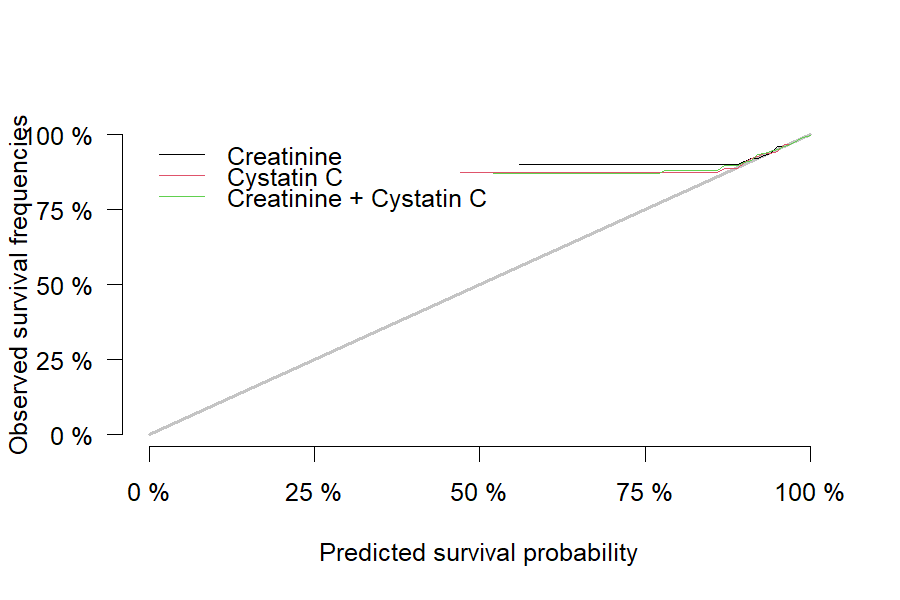
**
